# A Broad Profile of Co-Dominant Epitopes Shapes the Peripheral *Mycobacterium tuberculosis* Specific CD8+ T-Cell Immune Response in South African Patients with Active Tuberculosis

**DOI:** 10.1371/journal.pone.0058309

**Published:** 2013-03-26

**Authors:** Rebecca Axelsson-Robertson, André G. Loxton, Gerhard Walzl, Marthie M. Ehlers, Marleen M. Kock, Alimuddin Zumla, Markus Maeurer

**Affiliations:** 1 Department of Microbiology, Tumor and Cell Biology, Karolinska Institute, Stockholm, Sweden; 2 DST/NRF Centre of Excellence for Biomedical Tuberculosis Research and MRC Centre for Molecular and Cellular Biology, Division of Molecular Biology and Human Genetics, Department of Biomedical Sciences, Faculty of Health Sciences, Stellenbosch University, Stellenbosch, South Africa; 3 Department of Medical Microbiology, University of Pretoria/NHLS, Pretoria, South Africa; 4 Division of Infection and Immunity, University College London Medical School, London, United Kingdom; 5 Therapeutic Immunology (TIM), Department of Laboratory Medicine, Karolinska Institutet and CAST, Karolinska University Hospital, Stockholm, Sweden; University of Cape Town, South Africa

## Abstract

We studied major histocompatibility complex (MHC) class I peptide-presentation and nature of the antigen-specific CD8+ T-cell response from South African tuberculosis (TB) patients with active TB. 361 MHC class I binding epitopes were identified from three immunogenic TB proteins (ESAT-6 [Rv3875], Ag85B [Rv1886c], and TB10.4 [Rv0288], including amino acid variations for Rv0288, i.e., A10T, G13D, S27N, and A71S for MHC allotypes common in a South African population (e.g., human leukocyte antigen [HLA]-A*30, B*58, and C*07). Inter-allelic differences were identified regarding the broadness of the peptide-binding capacity. Mapping of frequencies of *Mycobacterium tuberculosis (M. tb)* antigen-specific CD8+ T-cells using 48 different multimers, including the newly constructed recombinant MHC class I alleles HLA-B*58:01 and C*0701, revealed a low frequency of CD8+ T-cell responses directed against a broad panel of co-dominant *M. tb* epitopes in the peripheral circulation of most patients. The antigen-specific responses were dominated by CD8+ T-cells with a precursor-like phenotype (CD45RA+CCR7+). The data show that the CD8+ T-cell response from patients with pulmonary TB (prior to treatment) is directed against subdominant epitopes derived from secreted and non-secreted *M. tb* antigens and that variant, natural occurring *M. tb* Rv0288 ligands, have a profound impact on T-cell recognition.

## Introduction

8.8 million incident tuberculosis (TB) cases and 1.45 million TB-related deaths were reported in 2010, with the highest TB rates occurring in sub-Saharan Africa [1]. The clinical outcome and presentation of TB is shaped by the interaction of *Mycobacterium tuberculosis* (*M. tb*) genetics and the host's immunological response [2].

CD8+ T-cells contribute to the cellular immune defense against TB [3] early during *M. tb* infection [4]. Activation and expansion of *M. tb-*specific and Major histocompatibility complex (MHC) class I restricted CD8+ T-cells requires the intracellular processing of *M. tb* antigens and subsequent presentation of epitopes by MHC class I cell-peptide complexes on the cell surface of antigen presenting cells. The immunogenicity of an *M. tb* epitope is influenced by several factors: i) the epitope must be generated and transported to the endoplasmic reticulum, by overcoming limitations in; cross-presentation [5] proteasome-dependent proteolysis [6] and the transporter associated with antigen processing [7]; ii) the epitope must bind to the MHC haplotype with appropriate affinity and dissociation rate; iii) the peptide-MHC (p-MHC) complex must then be recognized by an appropriate T-cell receptor (TCR) repertoire.

In order to determine immunogenicity of selected candidate *M. tb* epitopes, it is vital to correlate peptide MHC-binding to the actual detection of antigen-specific T-cells without *in vitro* manipulation (i.e. using *in vitro* expansion). This can be achieved in an unbiased way using MHC class I multimers loaded with molecularly defined pathogen-derived epitopes [8].

A marked heterogeneity characterizes the human immune-response against *M. tb*; the host-pathogen interplay dictates the outcome of infection. Different clustering of MHC alleles, human leukocyte antigens (HLA), are associated with geographic locations or ethnicity [9]. This association may, in part, reflect local host-pathogen co-adaptation which has driven both MHC and pathogen diversification. Differences in ethnicity and MHC alleles have previously been shown to play a role in T-cell responses to a number of infectious diseases, e.g. Hepatitis B [10], Hepatitis C [11] and human immunodeficiency virus (HIV) [12]. Associations between the host populations and defined lineages of *M. tb* have previously been identified [13], [14], therefore we chose to study the impact of different host (MHC class I alleles) and pathogen (strain-to-strain epitope) variations on *M. tb* peptide presentation on three levels: 1) peptide-MHC association, 2) TCR-recognition and 3) phenotype and cytotoxicity of CD8+ antigen-specific T-cells directed against different *M. tb* target antigens.

Up until now, only a few *M. tb* T-cell epitopes, restricted by a limited number of MHC class I alleles have been identified [15]. Therefore, there is a great need to identify additional *M. tb* epitopes restricted by a more diverse MHC class I allelic repertoire that reflects ethnical populations in Africa. In many regions of sub-Saharan Africa including South Africa, the most common HLA-A molecules are A*02, A*23, A*30 and A*68, the most common HLA-B alleles are B*15, B*42, B*53 and B*58 and the most common HLA*C alleles are C*04, C*06, C*07 and C*17 [16], [17], alleles for which only very few *M. tb* epitopes have previously been defined [15], [17].

Not only the MHC class I alleles show diversity, also the pathogen shows variations. It was previously thought that the *M. tb* genome is relatively stable, however an increasing number of strain-to strain differences, i.e. insertions, deletions and point mutations in the *M. tb* genome have been observed [18]. This genetic diversity might have both phenotypical and immunological impacts, e.g. variant T-cell epitopes may lead to different cellular immune response [19], [20]. Cellular immune responses directed to variant *M. tb* epitopes may also be responsible for the observation that mixed *M. tb* infections occur [21] and that TB patients are prone to re-infections [22]. Only a limited number of *M. tb* strains have been sequenced and most genetic variations appear to reside in non-essential *M. tb* genes. This appears to be true for many *M. tb* proteins, with the exception of the small immunogenic protein Rv0288. This protein has the following identified naturally occurring single amino acid exchanges: A10T, G13D, S27N and A71S [23]. All of these substitutions are part of previously reported CD8+ T-cell epitopes [17], [24] and might therefore interfere either with antigen presentation or subsequent T-cell recognition.

We chose to include 6 different *M. tb-*derived antigens in this study, since *M. tb* proteins can be differentially expressed or secreted during the course of infection. The immunodominant *secreted* TB proteins Ag85B (Rv1886c) and ESAT-6 (Rv3875) are part of several new TB vaccine candidates, [25], [26], [27]. They are early targets of cellular immune responses, and expressed at different time points of the infection, based on pre-clinical models [28], [29]. While Rv3875 (ESAT-6) is only expressed in *M. tb* and a few other mycobacterial species (e.g. *M. marinum* and *M. kansasii*) [30], Ag85B and TB10.4 (Rv0288), are, in addition to *M. tb*, expressed in the *Mycobacterium bovis* derived vaccine strain bacillus Calmette-Guérin (BCG) as well as in mycobacteria other than tuberculosis. The *non-secreted M. tb* proteins, glycosyl transferase (1) (Rv2958c), glycosyl transferase (2) (Rv2957) and cyclopropane-fatty-acid synthase (CFA synthase) (Rv0447c) are predominantly expressed in slow growing bacteria. They have previously been identified from our group as targets for humoral and cell-mediated immune responses [31], [32], [33]. These *M. tb* antigens are involved in cellular metabolism [34], [35], [36] and are producing factors associated with resistance to macrophage mediated killing [37].

We report in this study the detailed mapping of *M. tb* peptides to some of the most frequent MHC alleles in South Africa, their impact on T-cell recognition and T-cell homing/differentiation markers using a broad panel of *M. tb* MHC class I-peptide multimers.

## Materials and Methods

### Recombinant proteins

The heavy and light chains of HLA-A*02:01, A*24:02, A*30:01, A*30:02, A*68:01, B*07:02, B*58:01 and C*07:01 were cloned into bacterial expression vectors (pET24d+ and pHN1). The MHC molecules were produced as previously described [8], [38]; heavy and light chains were produced in *Escherichia coli* Bl21 DE3 pLys (Invitrogen, Carlsbad, California) as inclusion bodies, they were purified, solubilized and folded to correct trimeric structure in a pH 8.0 Tris-EDTA-arginine buffer (Sigma-Aldrich Sweden AB, Stockholm, Sweden) together with allele-specific candidate peptides (JPT Peptide Technologies GmbH, Berlin). The peptides were: A*02:01 – FLPSDFFPSV (HBV CORE), A*24:02 – RYLKDQQLL (HIV env), A*30:01 – KTKDIVNGL (F-actin capping protein beta), A*30:02 – KIQNFRVYY (HIV-integrase), A*68:01 – KTGGPIYKR (Influenza virus nucleoprotein), B*07:02 – TPRVTGGGAM (CMV pp65), B*58:01 – IAMESIVIW (HIV RT) and C*07:01 – KYFDEHYEY (CDC28 protein). Correctly folded trimeric p-MHC-complexes were then concentrated, biotinylated using the enzyme BirA (Avidity, Aurora, USA) and affinity purified using an avidin column (Thermo Fisher Scientific, Rockford, USA).

### Peptide screening assay

96-well plates coated with biotinylated-bovine serum albumin (BSA) and avidin (from BeckmanCoulter, San Diego, USA) were used to immobilize 0.5 µg/ml biotinylated monomers. Overlapping nonameric peptides (JPT Peptide Technologies GmbH, Berlin, Germany) covering the three TB proteins TB10.4 (Rv0288), Ag85B (Rv1886c) and ESAT-6 (Rv3875) were used in binding, affinity and off-rate studies as well as variant peptides from Rv0288. The duplicated experiments were performed as previously described [39], [40], shortly; the monomer-coated plates were stripped of the placeholder peptide leaving the heavy chain free to re-associate with an added candidate peptide after addition of *β*_2_-microglobulin. Positive binding was detected using a fluorescent labeled conformation-dependent antibody (A*30; IgM, clone 0273HA [One Lambda Inc, Canoga Park, USA], A*02, A*24, A*68, B*07, B*58 and C*07; fluorescein isothiocyanate (FITC)-conjugated anti-HLA-A, -B and –C [BeckmanCoulter, San Diego, USA]). The binding of each candidate peptide was compared to the binding of an appropriate control peptide (same peptides as used to produce the monomers, listed above). For selected peptides further characterization of the binding were made using affinity and off-rate assays. Affinity was measured by incubating titrated peptide concentrations (10−4 to 10−9 M) on the MHC coated 96-well plates in a manner similar to the binding assay. An ED_50_ value, i.e. the peptide quantity needed to achieve 50% binding saturation was calculated. By incubating the candidate peptide for different time points (0, 0.5 h, 1 h, 1.5 h, 2 h, 4 h, 6 h and 8 h), the stability of the p-MHC complexes could be assessed as a t_1/2_ value, which is defined as the time point when 50% of the initial peptide concentration has dissociated from the HLA-peptide molecule complexes. The binding, affinity, and off-rate-values were calculated using the *iTopia™* System Software (BeckmanCoulter, San Diego, USA). Sigmoidal dose response curves were generated using Prism® 4.0 (GraphPad).

### Selection of epitopes for multimer construction

Based on the data generated in the peptide binding assays, previously reported data [17], [24], [40], [41], [42], or high SYFPEITHI score [43], a set of 48 multimers covering epitopes from the TB proteins Rv0288, Rv1886c, Rv3875, Rv2957, Rv2958c and Rv0447c were obtained from BeckmanCoulter, San Diego, USA; from Immudex, Copenhagen, Denmark or produced in our lab as previously described [8].

### Patient data

27 patients diagnosed with active pulmonary TB (prior to any treatment), diagnosed with at least two positive sputum smears for acid-fast bacilli or positive sputum culture for *M. tb* were enrolled at the University of Stellenbosch, Cape Town and at the Pretoria Academic Hospital in South Africa. Demographic data of the patients are provided in Table S1. Peripheral blood mononuclear cells (PBMCs) were separated at each location after informed consent. The participants provided their written consent to participate in this study and these documents are on file at the clinical sites with the PI. The written consent forms were also submitted to the respective ethical committees and were approved. The institutional review boards (reference no. 45/2008, Faculty of Health Sciences, University of Pretoria, South Africa and no. N05/11/187, Health research and Ethics Committee, Stellenbosch University) approved the study. Frozen PBMCs were shipped to Sweden and HLA-typed using sequence-specific primer (SSP) typing kits (One Lambda Inc. Canoga Park, USA). Ethical consent from the ethical committee in Stockholm (ref. 2011/863-31/2) was acquired prior to sample analysis.

### Cellular analysis with multimers

Freshly thawed PBMCs were incubated with MHC class I multimers for 30 min at 37°C. Multimer positive events were recorded in the CD3+CD8+CD4− compartment using the following antibodies: anti-CD3-phycoerytin (PE)/Texas red (ECD) (clone: UCHT1) (BeckmanCoulter, San Diego, USA), anti-CD8α- allophycocyanin (APC)/Cy7 (clone: SK1) (Becton Dickinson, Franklin Lakes, USA) and anti-CD4-Pacific orange (clone: S3.5) (Invitrogen, Carlsbad, USA). The multimers were fluorescently labeled with streptavidin-PE, streptavidin-APC and fluorescein isothiocyanate (FITC). Cells in the CD3+CD8−CD4+ compartment were excluded from enumeration of CD3+CD8+multimer+ events. All cellular analyses were performed using a FACS Gallios flow-cytometer (BeckmanCoulter, San Diego, USA). Only multimer responses at least at least three times higher than the negative control and for which we could detect more than 50 events were further analyzed.

### Phenotypic analysis of the T-cells

Phenotypic analysis of antigen-specific cells was performed using the following Abs: anti-CD45RA-PerCP/Cy5.5 (Clone HI100) (Biolegend, San Diego, USA) and anti-CCR7-PE/Cy7 (Clone 3D12) (Becton Dickinson, Franklin Lakes, USA). Anti-CD107a-Pacific blue (PB) (clone: H4A3) (Biolegend, San Diego, USA) was used as a degranulation marker and analysis of IL-7Rα (alpha chain of the IL-7R) was performed using anti-CD127 APC/Alexa-700 (clone: R34.34) (BeckmanCoulter, San Diego, USA).

### Statistics

Statistical significance between different T-cell populations was evaluated using Prism® 4.0 (GraphPad Software, La Jolla, USA) using a Student's two-sided t-test.

## Results

### Peptide-MHC binding analysis – epitope identification

Overlapping peptides covering the amino acid sequences of the *M. tb* proteins Rv1886c (Ag85B) (n = 260) and Rv3875 (ESAT-6) (n = 87) were used to study individual peptide binding to 5 HLA-A MHC class I molecules (A*02:01, A*24:02, A*30:01, A*30:02 and A*68:01), 2 HLA-B alleles (B*07:02 and B*58:01) and 1 HLA-C allele (C*07:01). Peptides from Rv0288 (TB10.4) (n = 88) were used to study peptide binding to previously non-reported MHC class I alleles (HLA-A*68:01, B*58:01 and C*07:01). Peptides were regarded as positive binders if the binding exceeded >40% of the respective positive control peptide. In total, we could identify 361 MHC class I binding epitopes in the three proteins (Ag85B, TB10.4 and ESAT-6), of which 92% were previously not reported. 68 epitopes were associated with A*02:01, 43 associated with A*24:02, 8 with A*30:01, 99 with A*30:02, 43 with A*68:01, 25 with B*07:02, 45 with B*58:01 and 30 with C*07:01. In total, A*30:02 had the highest binding frequency (27%) followed by A*02:01 (19%), A*24:02 (12%), A*68:01 (12%), B*58:01 (12%), C*07:01 (8%), B*07:02 (7%) and A*30:01 (2%).

For Rv3875, we could identify 40 binding epitopes, of which more than half were associated with HLA-A*30:02 (n = 21), followed by A*02:01 (n = 11), A*68:01 (n = 3), A*24:02 (n = 2), B*58:01 (n = 2) and C*07:01 (n = 1). We were not able to identify any epitopes from Rv3875 binding to the alleles A*30:01 and B*07:02. In total, 34 peptides (39%) bound to at least one HLA allele and most of the binding peptides were located in the middle of the protein (Table 1).

**Table 1:** MHC class I binding, affinity and off-rate data for peptide-epitopes derived from Rv3875 (ESAT-6).

		A*02:01			A*24:02			A*30:01			A*30:02			A*68:01			B*07:02			B*58:01			C*07:01		
Peptide ID	Sequence*	Bind**	Aff	O-rate	Bind	Aff	O-rate	Bind	Aff	O-rate	Bind	Aff	O-rate	Bind	Aff	O-rate	Bind	Aff	O-rate	Bind	Aff	O-rate	Bind	Aff	O-rate
Rv3875_1–9_	MTEQQWNFA	13			0			0			**52**	2E-08	1.4	25			2			2			0		
Rv3875_4–12_	QQWNFAGIE	**45**	n.d	n.d	0			0			39			15			1			0			11		
Rv3875_12–20_	EAAASAIQG	0			0			0			0			**59**	8E-07	0.8	5			0			0		
Rv3875_16–24_	SAIQGNVTS	1			15			11			**67**	3E-08	0.9	29			4			12			12		
Rv3875_17-25_	*AIQGNVTSI*	**45**	n.d.	n.d.	9			0			**49**	n.d.	n.d.	29			4			21			14		
Rv3875_18–26_	IQGNVTSIH	**51**	1E-06	0.2	0			13			36			17			3			7			5		
Rv3875_21–29_	NVTSIHSLL	37			0			0			0			**67**	7E-09	0.6	3			0			6		
Rv3875_24–32_	SIHSLLDEG	1			38			29			**112**	2E-08	1.0	0			5			5			11		
Rv3875_25–33_	IHSLLDEGK	0			0			20			**46**	n.d.	n.d.	7			5			10			9		
Rv3875_27–35_	SLLDEGKQS	**54**	1E-06	1.4	0			12			0			10			3			1			4		
Rv3875_28–36_	LLDEGKQSL	**61**	7E-07	4.9	0			20			0			4			3			2			19		
Rv3875_35–43_	**SLTKLAAAW**	**47**	n.d.	n.d.	12			6			**50**	n.d.	n.d.	10			5			**48**	4E-07	0.9	8		
Rv3875_36-44_	LTKLAAAWG	15			0			13			0			21			6			**47**	7E-07	1.2	13		
Rv3875_38-46_	KLAAAWGGS	**57**	1E-07	2	33			0			39			0			6			21			11		
Rv3875_39-47_	*LAAAWGGSG*	8			4			14			**114**	7E-09	2.1	10			4			7			**120**	4E-08	1.0
Rv3875_40-48_	AAAWGGSGS	8			0			29			**129**	5E-09	1.1	15			4			8			29		
Rv3875_41–49_	AAWGGSGSE	29			2			18			**167**	3E-07	1.5	29			4			9			29		
Rv3875_42–50_	AWGGSGSEA	4			4			16			**90**	8E-07	1.7	11			6			6			29		
Rv3875_43–51_	WGGSGSEAY	3			0			18			**89**	4E-07	1.1	2			7			2			21		
Rv3875_46–54_	SGSEAYQGV	22			**54**	9E-09	0.5	0			0			0			9			2			24		
Rv3875_49–57_	EAYQGVQQK	0			0			0			0			**74**	4E-08	7.5	10			0			0		
Rv3875_50–58_	AYQGVQQKW	0			**60**	1E-08	0.4	0			33			6			10			35			0		
Rv3875_53–61_	GVQQKWDAT	0			0			9			**78**	3E-08	1.3	0			10			6			9		
Rv3875_54–62_	*VQQKWDATA*	**54**	5E-09	1.2	0			21			**76**	6E-08	1.6	0			9			12			9		
Rv3875_55–63_	QQKWDATAT	0			0			29			**60**	9E-08	1.7	9			9			8			4		
Rv3875_60–68_	ATATELNNA	31			0			0			**67**	3E-07	3.8	0			12			0			0		
Rv3875_61–69_	TATELNNAL	11			15			4			**106**	9E-09	2.6	13			12			0			5		
Rv3875_62–70_	ATELNNALQ	8			13			16			**45**	n.d.	n.d.	5			12			4			8		
Rv3875_68–76_	ALQNLARTI	**73**	3E-08	1.7	0			0			17			0			8			0			0		
Rv3875_69–77_	LQNLARTIS	27			26			11			**77**	1E-08	2.1	8			10			20			3		
Rv3875_71–79_	NLARTISEA	**72**	4E-08	2.3	11			15			17			9			10			21			12		
Rv3875_74–82_	RTISEAGQA	18			0			19			**98**	3E-08	2	4			11			34			8		
Rv3875_79–87_	AGQAMASTE	0			0			20			**58**	5E-09	2.3	13			9			11			28		
Rv3875_82–90_	*AMASTEGNV*	**87**	3E-08	1.0	0			0			**102**	7E-09	2.5	0			11			0			3		

* Promiscuous epitopes are marked in bold or italic (italic – binding >1 allele, bold – binding to >2 alleles). **Positive binding epitopes are marked in bold. Binding is reported as percent relative the binding of a positive control peptide, affinity is reported as an ED50 value (M), and off-rate is reported as a *t*_1/2_ value (h), as described in material and methods. Multimer MHC-peptide complexes were constructed for the epitopes: A02-Rv3875_LLDEGKQSL_, A02-Rv3875_AMASTEGNV_, A24-Rv3875_AYQGVQQKW_ and A3002-Rv3875_AMASTEGNV_.

**Figure 1:**
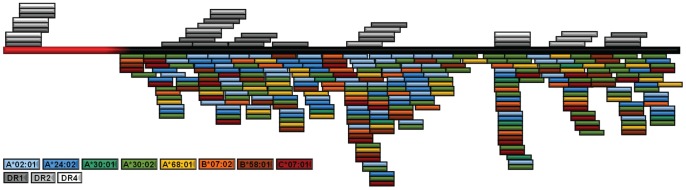
Alignment of the major histocompatibility complex (MHC) class I-binding epitopes with the amino acid sequence of Rv1886c. Epitopes identified for HLA-A*02:01 are shown in light blue, for A*24:02 in dark blue, for A*30:01 in dark green, peptides for A*30:02 in light green, for A*68:01 in yellow, for B*07:02 in orange, for B*58:01 in brown and for C*07:01 in red. Alignments of MHC class II restricted epitopes (HLA-DR1 – dark grey, DR2 – medium grey and DR4 – light grey) that have previously been reported in reference [32] are included as well. The signal sequence of Rv1886c is shown in red and the rest of the protein in black.

For the much larger *M. tb* protein Rv1886c, 292 epitopes could be identified associating with the following alleles: A*02:01 (n = 57), A*24:02 (n = 41), A*30:01 (n = 8), A*30:02 (n = 78), A*68:01 (n = 29), B*07:02 (n = 25), B*58:01 (n = 29) and C*07:01 (n = 25). The epitopes were scattered throughout the amino acid sequence with some clustering of promiscuous epitopes in the middle. 155 peptides (60%) bound to at least one MHC class I allele; 75 epitopes were found to be promiscuous, i.e. binding to more than one MHC class I allele. Some were highly promiscuous and cross-binding to 7 out of 8 MHC class I alleles (VANNTRLWV [Rv1886c_241–249_] [A*02:01, A*24:02, A*30:01, A*30:02, B*07:02, B*58:01 and C*07:01]) or 6 out of 8 (KLVANNTRL [Rv1886c_239–247_] [A*02:01, A*24:02, A*30:01, A*30:02, B*07:02 and B*58:01]) (Figure 1 and Table S2).

In addition, we could identify 29 binding epitopes from Rv0288 associating with A*68:01 (n = 11), B*58:01 (n = 14) and C*07:01 (n = 4) (Table 2). We also wanted to elucidate the effect of naturally occurring single amino acid substitutions within these epitopes concerning the binding to different MHC class I molecules. The reported substitutions were part of 18 previously identified binding epitopes (from the sequence of H37Rv) and we compared the binding-rate of these *M. tb* epitopes to 24 novel epitopes which were constructed including one or two amino acid substitutions (Table 3). The variant epitope showed better MHC class I-binding as compared to the reference epitopes in only 13% of the tested peptide-MHC binding combinations. All of these were restricted by ‘promiscuous alleles’ like HLA-A*02:01, A*24:02 and A*30:02. Changing a binding epitope restricted by alleles with intermediate (A*68:01 and B*58:01) or a more restricted binding pattern (A*30:01, B*07:02 and C*07:01) resulted in a profound decrease of peptide binding or even total abrogated MHC class I-peptide binding.

**Table 2:** MHC class I binding, affinity and off-rate data for peptide-epitopes derived from Rv0288 (TB10.4).

		A*68:01			B*58:01			C*07:01		
Peptide ID	Sequence*	Bind**	Aff	O-rate	Bind	Aff	O-rate	Bind	Aff	O-rate
Rv0288_1–9_	MSQIMYNYP	29			**57**	4E-06	1.1	10		
Rv0288_4–12_	*IMYNYPAML*	0			**110**	2E-07	1.0	**67**	2E-07	0.2
Rv0288_6–14_	YNYPAMLGH	30			0			**48**	7E-06	0.3
Rv0288_35–43_	*EQAALQSAW*	**47**	1E-05	2.8	**79**	6E-06	0.8	0		
Rv0288_36–44_	QAALQSAWQ	17			**62**	5E-05	0.5	7		
Rv0288_41–49_	SAWQGDTGI	30			**46**	3E-08	0.6	12		
Rv0288_43–51_	WQGDTGITY	**61**	1E-05	6.0	33	8E-06	1.8	0		
Rv0288_46–54_	DTGITYQAW	7			**41**	1E-06	2.3	0		
Rv0288_47–55_	TGITYQAWQ	8			**40**	4E-06	0.8	0		
Rv0288_48–56_	GITYQAWQA	0			**46**	9E-08	0.2	0		
Rv0288_49–57_	*ITYQAWQAQ*	**56**	9E-06	1.9	**78**	2E-07	0.5	0		
Rv0288_50–58_	TYQAWQAQW	0			**119**	1E-07	1.0	0		
Rv0288_51–59_	YQAWQAQWN	23			**52**	2E-06	2.0	0		
Rv0288_52–60_	*QAWQAQWNQ*	**55**	7E-06	3.0	**45**	4E-06	0.5	0		
Rv0288_61–69_	AMEDLVRAY	0			14			**46**	5E-06	0.1
Rv0288_66–74_	VRAYHAMSS	29			10			**79**	1E-06	0.2
Rv0288_70–78_	HAMSSTHEA	**77**	6E-06	0.5	16			6		
Rv0288_73–81_	SSTHEANTM	0			**41**	2E-06	0.8	7		
Rv0288_77–85_	EANTMAMMA	**83**	7E-04	1.9	0			3		
Rv0288_78–86_	ANTMAMMAR	**147**	3E-06	2.4	0			12		
Rv0288_79–87_	NTMAMMARD	**67**	7E-05	2.3	0			0		
Rv0288_80–88_	TMAMMARDT	**47**	6E-05	2.7	0			0		
Rv0288_81–89_	MAMMARDTA	**96**	5E-06	2.0	23			19		
Rv0288_86–94_	*RDTAEAAKW*	**65**	1E-05	4.3	**45**	5E-06	2.6	0		

* Promiscuous epitopes are marked in italic. **Positive binding epitopes are marked in bold. Binding is reported as percent relative the binding of a positive control peptide, affinity is reported as an ED50 value (M), and off-rate is reported as a *t*_1/2_ value (h), as described in material and methods. MHC class I-peptide complexes were constructed for the epitopes: A68-Rv0288_HAMSSTHEA_ and A68-Rv0288_ANTMAMMAR_.

**Table 3:** MHC class I binding, affinity and off-rate data for the variant peptide-epitopes derived from Rv0288 (TB10.4).

		A*02:01			A*24:02			A*30:01			A*30:02			A*68:01			B*07:02			B*58:01			C*07:01		
ID	Sequencê	Bindˆ?	Aff	O-rate	Bind	Aff	O-rate	Bind	Aff	O-rate	Bind	Aff	O-rate	Bind	Aff	O-rate	Bind	Aff	O-rate	Bind	Aff	O-rate	Bind	Aff	O-rate
Rv0288_2–10wt_	SQIMYNYPA	78*	4E-07*	4.9*							46**	2E-06	0.3												
Rv0288_2–10m1_	SQIMYNYP***T***	**47**	1E-06	0.7							31	4E-06	0.5												
Rv0288_3–11wt_	QIMYNYPAM	68*	1E-06	1.1	55*	1E-06	0.1	163**	4E-06	0.8	90**	4E-08	1.1				28*	8E-04	1.5	34	1E-05	0.4			
Rv0288_3–11m1_	QIMYNYP***T***M	**51**	3E-06	0.7	**40**	2E-06	0.5	12	n.d.	n.d.	23	n.d.	n.d.				5	n.d.	n.d.	9	n.d.	n.d.			
Rv0288_4–12wt_	IMYNYPAML	94*	8E-07	26.9	107*	1E-07	1.6	146**	5E-06	0.5	91**	8E-09	3.1				35*	3E-05	1.6	**110**	2E-07	1.0	**67**	2E-07	0.2
Rv0288_4–12m1_	IMYNYP***T***ML	**88**	4E-07	4	**43**	8E-07	0.5	28	n.d.	n.d.	15	n.d.	n.d.				7	n.d.	n.d.	37	3E-04	0,5	5	n.d.	n.d.
Rv0288_5–13wt_	MYNYPAMLG				72*	6E-07	1.4				45**	7E-06	0.6												
Rv0288_5–13m1_	MYNYP***T***MLG				**47**	2E-07	0.5				23	n.d.	n.d.												
Rv0288_5–13m2_	MYNYPAML***D***				**60**	3E-08	0.4				**59**	2E-06	0.7												
Rv0288_5–13m3_	MYNYP***T***ML***D***				**76**	2E-08	0.5				0	n.d.	n.d.												
Rv0288_6–14wt_	YNYPAMLGH																						**48**	7E-06	0.3
Rv0288_6–14m1_	YNYP***T***MLGH																						0	n.d.	n.d.
Rv0288_6–14m2_	YNYPAML***D***H																						0	n.d.	n.d.
Rv0288_6–14m3_	YNYP***T***ML***D***H																						0	n.d.	n.d.
Rv0288_10–18wt_	AMLGHAGDM	46*	6E-06	1.7	25*	1E-04	3.1				97**	5E-08	1.2												
Rv0288_10–18m1_	***T***MLGHAGDM	10	n.d.	n.d.	4	n.d.	n.d.				**99**	5E-06	0.6												
Rv0288_10–18m2_	AML***D***HAGDM	**47**	8E-08	1.1	13	n.d.	n.d.				**100**	7E-07	0.4												
Rv0288_10–18m3_	**T**ML***D***HAGDM	24	n.d.	n.d.	14	n.d.	n.d.				0	n.d.	n.d.												
Rv0288_11–19wt_	MLGHAGDMA	67*	7E-07	1.4																					
Rv0288_11–19m1_	ML***D***HAGDMA	**48**	6E-08	1.5																					
Rv0288_13–21wt_	GHAGDMAGY										71**	4E-08	1.3												
Rv0288_13–21m1_	**D**HAGDMAGY										**141**	1E-08	0.3												
Rv0288_20–28wt_	GYAGTLQSL				76*	3E-07	3.7																		
Rv0288_20–28m1_	GYAGTLQ***N***L				**68**	1E-07	0.3																		
Rv0288_24–32wt_	TLQSLGAEI	46*	3E-06	1.7	16*	9E-08	39.8																		
Rv0288_24–32m1_	TLQ***N***LGAEI	26	n.d.	n.d.	4	n.d.	n.d.																		
Rv0288_26–34wt_	QSLGAEIAV	48*	2E-06	0,9																30	2E-04	0.1			
Rv0288_26–34m1_	Q***N***LGAEIAV	28	n.d.	n.d.																0	n.d.	n.d.			
Rv0288_27–35wt_	SLGAEIAVE	28*	1E-05	1.8																					
Rv0288_27–35m1_	**N**LGAEIAVE	29	n.d.	n.d.																					
Rv0288_64–72wt_	DLVRAYHAM				31*	2E-06	2.9							34	4E-04	3.3									
Rv0288_64–72m1_	DLVRAYH***S***M				0	n.d.	n.d.							4	n.d.	n.d.									
Rv0288_65–73wt_	LVRAYHAMS							158**	2E-06	1.8	75**	2E-06	1.0												
Rv0288_65–73m1_	LVRAYH***S***MS							7	n.d.	n.d.	30	1E-08	0.9												
Rv0288_66–74wt_	VRAYHAMSS																						**79**	1E-06	0.2
Rv0288_66–74m1_	VRAYH***S***MSS																						3	n.d.	n.d.
Rv0288_67–75wt_	RAYHAMSST										73**	1E-06	1.2				39*	2E-05	0.8	39	1E-05	0.2	**35**	1E-05	0.2
Rv0288_67–75m1_	RAYH**S**MSST										7	n.d.	n.d.				14	n.d.	n.d.	32	2E-08	0.4	0	n.d.	n.d.
Rv0288_68–76wt_	AYHAMSSTH										70**	3E-07	1.2												
Rv0288_68–76m1_	AYH***S***MSSTH										**70**	2E-07	0.5												
Rv0288_70–78wt_	HAMSSTHEA	43*	6E-06	2.5							88**	3E-07	0.6	**77**	6E-06	0.5									
Rv0288_70–78m1_	H***S***MSSTHEA	**45**	1E-03	0.3							39	6E-09	0.9	0	n.d.	n.d.									

?Amino acid substitutions within an epitope are marked in bold italic text. *??*Positive binding epitopes are marked in bold. Binding is reported as percent relative the binding of a positive control peptide, affinity is reported as an ED50 value (M), and off-rate is reported as a *t*_1/2_ value (h), as described in material and methods. MHC class I-peptide complexes were constructed for the epitopes: A02-Rv0288_AMLGHAGDM_, A02-Rv0288_AML***D***HAGDM_, A02-Rv0288_MLGHAGDMA,_ A02-Rv0288_ML***D***HAGDMA_, A24-Rv0288_MYNYPAMLG_ and A24-Rv0288_MYNYP***T***ML***D***_. *Binding, affinity and off-rate have previously been reported in reference [24]. **Binding, affinity and off-rate have previously been reported in reference [17].

### Analysis of the nature of the p-MHC binding

Next, we analyzed in greater detail the Rv3875 epitopes that bound with more than 50% to the respective MHC class I test molecules as compared to the positive control peptide (30 epitopes). The nature of peptide binding to an MHC class I molecule can be divided into affinity, i.e. the binding strength between the peptide and the MHC molecule, and the dissociation rate, i.e. the off-rate (t_1/2_) of the peptide-MHC complex. The affinity ranged between 1 μM and 5 nM with the majority of the peptides exhibiting an affinity in the lower end of the interval. The dissociation rate ranged between 0.2 h and 4.9 h; both extremes applied for HLA-A*02:01 (Table 1).

Since we identified a considerable number of binding epitopes from the protein Rv1886c, we analyzed the binding characteristics of only a selected set of peptides. The peptides were chosen based on 1) not being previously reported, 2) high binding compared to the positive control and 3) promiscuity (i.e. binding to several MHC molecules). In total, we analyzed affinity and dissociation rate from 121 novel *M. tb* candidate epitopes. The affinity ranged between 400 μM and 5 nM with the majority between 9 μM and 10 nM. Some of the peptides showed a very rapid dissociation rate (<0.1 h), while some formed a stable MHC class I-peptide complex for up to 8 hours. We identified a high range of intra-allelic differences (different peptides bound different to the same HLA allele) as well as inter-allelic differences (same peptides bound different to different alleles). The peptides bound generally in a more stable fashion to the HLA-A alleles as compared to the B and C alleles , with the exception of HLA-A*30:01 which exhibited an off-rate of less than 1 h. Also, peptides binding to alleles with a limited set of binding epitopes tended to dissociate more rapidly (Table S2).

We identified in total 29 novel epitopes from the protein Rv0288 binding to the alleles HLA-A*6801, B*58:01 and C*07:01 and analyzed the binding characteristics for the entire peptide set. The affinity ranged between 700 μM and 30 nM, and the off-rate between 0.1 h and 6 h. We could not see any specific inter- or intra-allelic differences regarding affinity but we could detect a very rapid dissociation rate of less than 20 minutes regarding all candidate epitopes binding to C*07:01 (Table 2).

Alterations in individual amino acids lead to an increased or decreased binding strength in an almost equal number of cases. However we were only able to measure affinity in 20 out of 53 cases due to that too low MHC-peptide binding makes it impossible to reliably study affinity and off-rate. This was the case for all the variant epitopes restricted by A*30:01, A*68:01, B*07:02 and C*07:01. In most cases, where the peptide variation resulted in increased affinity, it increased the total peptide binding. In cases where the MHC class I-peptide stability increased, the increase was only marginal. In general, the amino acid exchange lead to decreased MHC binding that could be explained by either a decrease in 1) binding strength and/or 2) stability of the MHC class I/peptide complex. However, a few outliers were identified for which we could see an increase in both parameters (binding strength and affinity) while the total peptide binding decreased, e.g. H***S***MSSTHEA (Rv0288_70–78_) to A*30:02 and ML***D***HAGDMA (Rv0288_11–19_) to A*02:01.

### Multimeric analysis of selected TB epitopes

A*02, A*24, A*30 A*68, B*07, B*58 and C*07 belong to the most prevalent MHC class I alleles in a South African population. Therefore, we constructed 33 different multimers from the *M. tb* proteins ESAT-6 (Rv3875), Ag85B (Rv1886c) and TB10.4 (Rv0288) as well as 12 multimers from other (non-secreted) *M. tb* antigens (e.g. glycosyl transferase 1 [Rv2958c], glycosyl transferase 2 [Rv2957] and CFA synthase [Rv0447c]) with the intention to evaluate the frequency, phenotype and functionality of antigen-specific T-cells. MHC-peptide multimers were used to enumerate CD8+ T-cell responses in blood from 27 patients diagnosed with active pulmonary TB in South Africa. Examples of staining and the gating strategy are provided in Figures S1 and S2.

**Figure 2:**
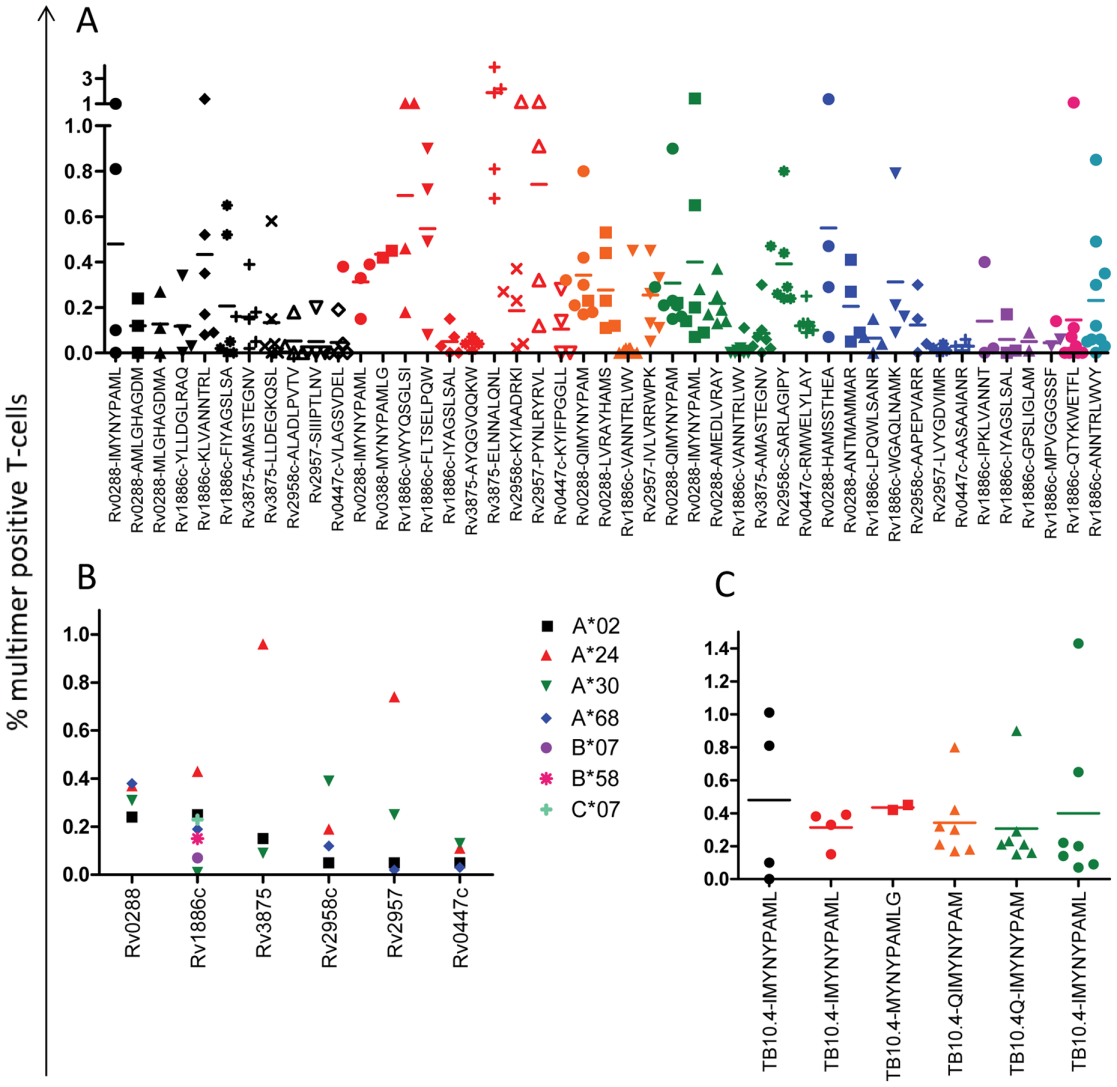
Percentage multimer positive CD8+ T-cells divided into different compartments. (A) Percentage antigen-specific recognition of the 45 individual multimers used in this study, each dot represents the staining in PBMCs from one patient and the different restricting alleles are shown in different colors (HLA-A*02:01 – black, A*24:02 – red, A*30:01 – orange, A*30:02 – green, A*68:01 – blue, B*07:02 – purple, B*58:01 – pink and C*07:01 – turquoise), each individual multimer within an allele is shown by different shapes of dots. (B) Average detection in all patients of antigen-specific T-cells divided per antigenic protein and per restricting allele (A*02:01 – black squares, A*24:02 – red triangles, A*30:01/A*30:02 – green triangles, A*68:01 – blue diamonds, B*07:02 – purple circles, B*58:01 – pink crosses and C*07:01 – turquoise crosses). (C) Individual detection of antigen-specific T-cells specific for the ‘super-epitope’ (QI)MYNYPAM(LG), each dot represents the staining in an individual patient, different colors represents the different restricting allele (A*02:01 – black, A*24:02 – red, A*30:01 – orange and A*30:02 – green).

The number of antigen-specific T-cell frequencies ranged between 0 and 3.9% in the CD8+ T-cell population, with an average frequency of 0.3% multimer-reactive T-cells in CD8+ T-cells. We were able to identify inter-epitope specific variations that could be divided into 3 groups based on recognition frequencies. The first group included epitopes that were almost never recognized above the negative control multimer (e.g. A2-Rv0447c_VLAGSVDEL_, A3001-Rv1886c_VANNTRLWV_ and A3002-Rv1886c_VANNTRLWV_). The second group constituted epitopes for which we could detect specific T-cells only in some patients (e.g. A2-Rv3875_LLDEGKQSL_, A24-Rv2958c_KYIAADRKI_ and B58-Rv1886cQTYKWETFL). The third group contained epitopes that were recognized in blood from all patients (e.g. A2-1886c_KLVANNTRL_, A24-Rv3875_ELNNALQNL_ and A3002-Rv0288_QIMYNYPAM_) (Figure 2A and Table S3). Based on the construction of several different allele-specific multimers (e.g. 11 for HLA-A*02, 10 for HLA-A*24 and 11 for HLA-A*30), we could identify differences in the allele-specific repertoires. Some epitopes were never recognized by T-cells in any individuals, while others were recognized in PBMCs from all patients, such as the A*02-restricted epitope KLVANNTRL from Rv1886c, indicating immunodominance (Figure S3).

We could also detect general allele-specific variations in the detection of antigen-specific T-cells. Rv3875 epitopes were, for example, generally recognized very strongly by A*24-restricted T-cells, due to the high frequent recognition of the immunodominant epitope Rv3875_ELNNALQNL_. On the other hand, Rv2957 derived epitopes were not recognized in blood from HLA-A*02 and A*68 positive individuals at least the single epitope included in this study derived from this protein (Figure 2B).

Since we managed to produce 6 different multimers presenting the *M. tb* variant epitope (QI)MYNYPAM(LG), we were also able to examine the nature of antigen-specific T-cell recognition of ‘MHC-promiscuous epitopes’. We could not detect significant differences in the detection of antigen-specific T-cells, neither inter-allelic, i.e. if the same epitope IMYNYPAML was presented via the different HLA alleles (e.g. A*02, A*24 and A*30:02), nor intra-allelic when the almost identical epitopes was restricted by the same allele, e.g. A*24 (IMYNYPAML), MYNYPAMLG [0.3–0.4%]), and A*30:02 (QIMYNYPAM and IMYNYPAML [0.3–0.4%]). These data may suggest non-allele-specific T-cell recognition of ‘promiscuous epitopes’ like (I)MYNYPAML(G) (Figure 2C and Table S3), at least in the study population examined in this report.

### Phenotypic analysis of antigen-specific T-cells

**Figure 3:**
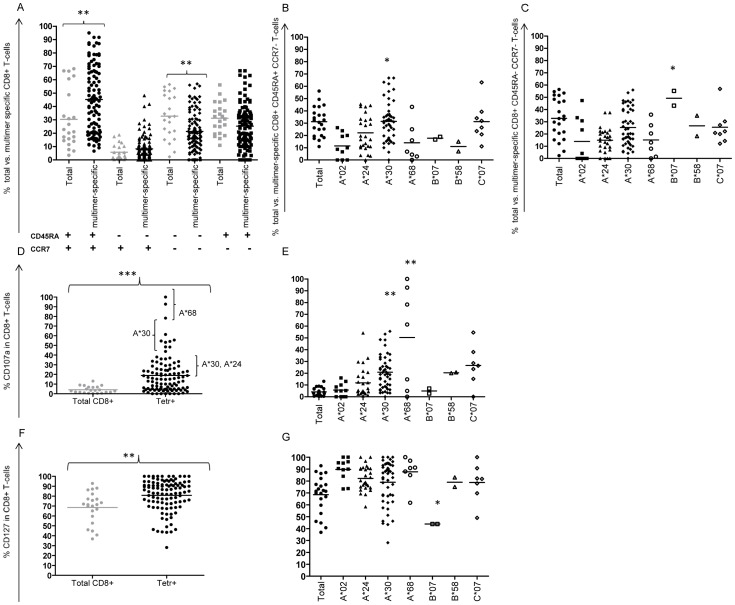
Frequencies of total CD8+ T-cells as well as different compartments of antigen-specific CD8+ T-cells expressing differentiation and maturation markers. (A) Total CD8+ T-cells (grey) vs. antigen-specific CD8+ T-cells (black) belonging to the different phenotypic compartments; naïve/precursor (CD45RA+CCR7+) (circles), central memory (CD45RA−CCR7+) (triangles), effector memory (CD45RA−CCR7−) (diamonds) and terminally differentiated cells (CD45RA+CCR7−) (squares). (B) Total CD8+ and antigen-specific T-cells belonging to the terminally differentiated compartment (CD45RA+CCR7−) and (C) effector memory compartment (CD45RA−CCR7−) divided per restricting MHC class I allele (Total CD8+ T-cells – filled circles, A*02:01 – filled squares, A*24:02 – filled triangles, A*30:01/A*30:02 – filled diamonds, A*68:01 – open circles, B*07:02 – open squares, B*58:01 – open triangles and C*07:01 – open diamonds). (D) Expression of the degranulation marker CD107a in total CD8+ T-cells (grey) vs. antigen-specific CD8+ T-cells (black). (E) Total CD8+ and antigen-specific T-cells expressing CD107a divided per restricting MHC class I allele (total CD8+ T-cells – filled circles, A*02:01 – filled squares, A*24:02 – filled triangles, A*30:01/A*30:02 – filled diamonds, A*68:01 – open circles, B*07:02 – open squares, B*58:01 – open triangles and C*07:01 – open diamonds). (F) Expression of the survival marker CD127 in total CD8+ T-cells (grey) vs. antigen-specific CD8+ T-cells (black). (G) Total CD8+ and antigen-specific T-cells expressing CD127 divided per restricting MHC class I allele (total CD8+ T-cells – filled circles, A*02:01 – filled squares, A*24:02 – filled triangles, A*30:01/A*30:02 – filled diamonds, A*68:01 – open circles, B*07:02 – open squares, B*58:01 – open triangles and C*07:01 – open diamonds). Each dot represents an individual multimer in one individual TB patient. Student's two-sided t-test was performed and significant values were calculated based on the following p-values: *p<0.05, **p<0.01, ***p<0.001.

Since we detected differences in epitope/antigen recognition based on the restricting MHC class I allele and in the numbers of antigen-specific T-cells, we proceeded to analyze the phenotype of *M. tb* antigen-specific cells. The phenotype of CD8+ T-cells can be defined by the cell-surface markers CD45RA and CCR7 [44], [45]. The phenotype frequency analysis revealed that precursor (CD45RA+CCR7+), effector memory (CD45RA−CCR7−) and terminally differentiated (CD45RA+CCR7−) are almost equally distributed (∼30% in each population), while the average percentage of central memory cells (CD45RA−CCR7+) was around 6%. On the other hand, we detected a significantly higher number of antigen-specific CD8+ T-cells in the precursor compartment (45%) (p = 0.01) as well as a lower frequency of cells in the effector memory compartment (21%) (p = 0.002) (Figure 3A).

We were able to detect allele-specific differences concerning T-cell maturation and differentiation. The HLA-A*30-restricted (*M. tb* specific) T-cells were found, to a higher extent, in the terminally differentiated effector compartment (p = 0.02) as compared to the other HLA-A restricted antigen-specific T-cells. A similar trend could be seen for the allele HLA-C*07 (Figure 3B). For effector memory antigen-specific CD8+ T-cells, we could detect an MHC class I allele family based difference with a significantly higher proportion of T-cells restricted by the allele HLA-B*07 and a similar trend for the other HLA-B alleles included in this study, i.e. B*58 (Figure 3C). For the other two phenotypic compartments (precursor and central memory T-cells), we could also identify a trend towards allele-specific differences, although none were significant (Figure S4) Comparison of differences within the phenotype of antigen-specific CD8+ T-cell between different *M. tb* proteins showed a profile independent of the nature of the *M. tb* antigens included in this study (Figure S4) and the same was found to be true for the different epitopes of the Rv0288 hot-spot (QI)MYNYPAM(LG) (Figure S5): differences in antigen-specific CD8+ T-cell maturation/differentiation were associated with the restricting MHC allele, yet not the presented *M. tb* epitope.

### Analysis of degranulation and survival markers

We analyzed the degranulation marker CD107a (LAMP-1), which correlates with the cytotoxic capacity of CD8+ T-cells. We compared the frequency of total CD8+ T-cells and the *M. tb* antigen-specific cells concerning CD107a expression and could detect significantly higher frequencies of antigen-specific T-cells expressing the degranulation marker CD107a (4% vs. 19%, p = 0.0005) (Figure 3D). The increase in CD107a expression was independent of the restricting MHC allele and the nature of the *M. tb* antigen and ranged from 5% (B*07) to 50% (A*68). *M. tb* epitopes could be divided into 4 groups according to the frequency of the antigen-specific CD8+ T-cells expressing CD107a. To the first epitope group belonged T-cells with more than 70% expressing CD107a, i.e. predominantly A*68 restricted epitopes. The second group consisted of epitopes recognized by cells expressing CD107a on 40–70% of the cells, i.e. mostly A*30-restricted epitopes. The third group consisted of epitopes recognized by cells expressing CD107a on 20–40% of the antigen-specific T-cells, i.e. mostly A*30 and A*24 restricted epitopes, and the rest belonged to the forth group. Generally, the HLA-A*30 and A*68 restricted antigen-specific T-cells expressed higher frequencies of CD107a (Figure 3E).

An important marker for T-cell survival is CD127 (IL-7 receptor α-chain). We were able to detect a significantly increased expression of this cell-surface marker on antigen-specific T-cells (81%) as compared to the total CD8+ T-cells (69%) (p = 0.001) (Figure 3F). This was also true for all MHC class I alleles presenting *M. tb* epitopes to CD8+ T-cells, except B*07 (Figure 3G). Based on variations between epitope-derived *M. tb* protein targets, we could neither detect difference regarding expression of CD107a nor of CD127 (Figure S6). This was also true when comparing the Rv0288 derived ‘super-epitope’ which binds to a higher number of MHC class I molecules (QI)MYNYPAM(LG) (Figure S6) Also here, the expression of CD107a and CD127 was closely associated with the MHC class I restricting allele, and not the *M. tb* target protein.

### Effect of amino acid substitutions on TCR recognition

**Figure 4:**
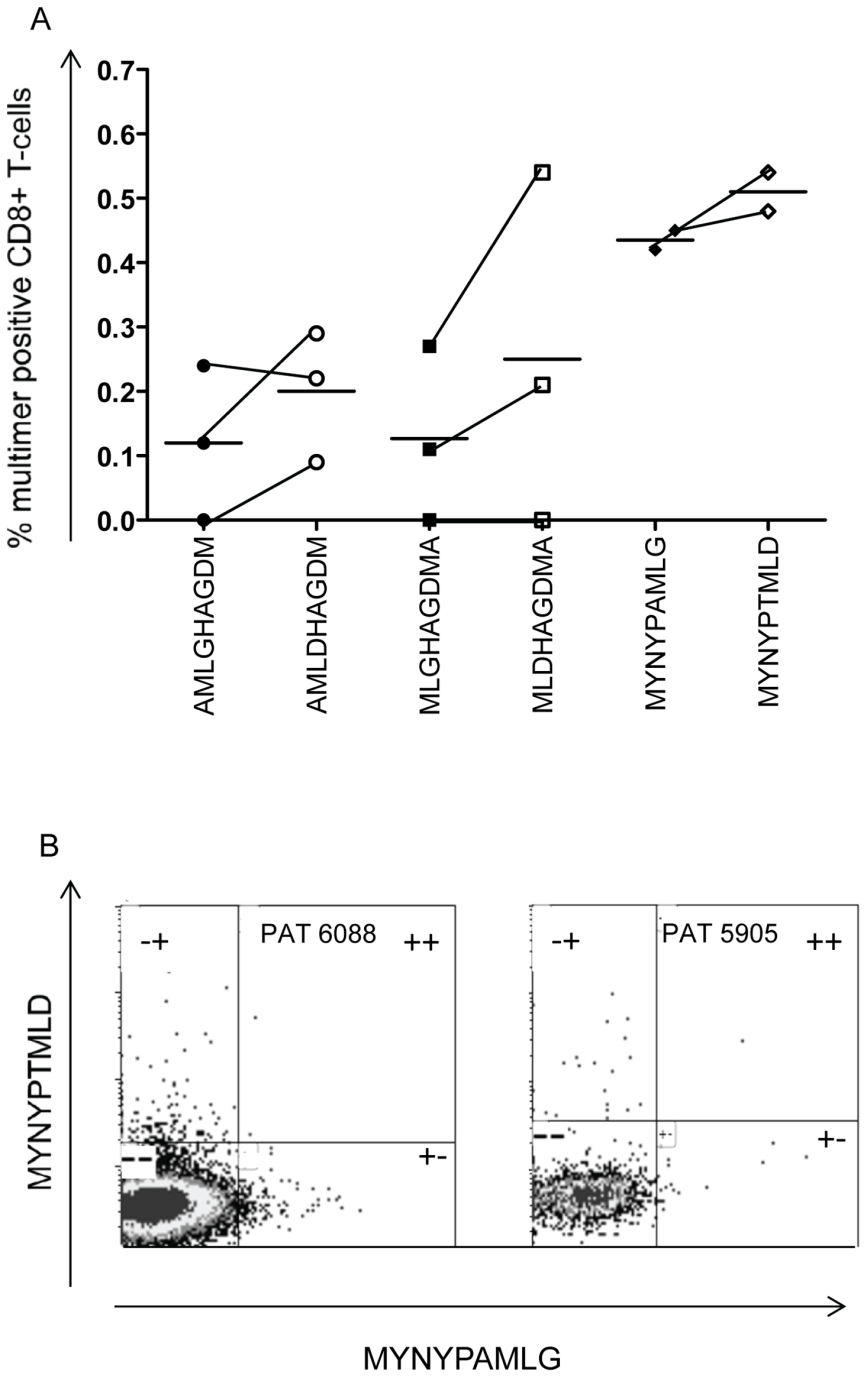
Antigen-specific recognition of the immunogenic epitopes from the H37Rv reference strain as well as the same epitopes containing naturally occurring amino acid substitutions. (A) Percent antigen specific CD8+ T-cell recognizing the A*02:01 restricted epitopes (AML(G/**D**)HAGDM) (circles) and (ML(G/**D**)HAGDMA) (squares) and the A*24:02 restricted epitope (MYNYP(A/**T**)ML(G/**D**) (diamonds), wild-type epitopes (closed symbols) and variant epitopes (open symbols). (B) Representative figures of multimer staining regarding the A*24:02 restricted epitope (MYNYP(A/**T**)ML(G/**D**)). The x-axis shows the staining of the wt epitope and the y-axis staining the variant epitopes. Numbers of events for the wt/mut epitopes regarding patient 6088 were +– 162, –+ 181 and ++ 2. A similar T-cell distribution could be detected regarding patient 5905 (+– 17, –+ 21 and ++ 1).

An amino acid substitution within an epitope might abrogate or profoundly change the interaction to a specific MHC class I allele. However, the effect on TCR recognition is biologically equally relevant. To be able to study TCR-MHC-class I peptide interaction, we constructed 3 pairs of multimers consisting of a wild-type *M. tb* target epitope (from H37Rv) and epitopes containing one or two naturally occurring substitutions for the alleles A*02 and A*24. The two epitopes binding to HLA-A*02 (AML(G/***D***)HAGDM and ML(G/***D***)HAGDMA) had the mutations in the 3^rd^ or the 4^th^ amino acid potentially being part of the TCR recognition site, while the epitope associating with A*24 (MYNYP(A/***T***)ML(G/***D***)) contained two mutations in the 6^th^ and 9^th^ position (of which the first position might affect TCR recognition). Although the association of these variant *M. tb* epitopes to its corresponding MHC molecule had either no effect, or showed *decreased* MHC binding, multimers containing these *M. tb* variant epitopes showed a tendency of detecting a higher frequency of antigen-specific T-cells as compared to multimers presenting the wild-type epitopes (A2_AML(G/***D***)HAGDM_ 0.12/0.20%, A2_ML(G/***D***)HAGDMA_ 0.13/0.25% and A24_MYNYP(A/***T***)ML(G/***D***)_ 0.44/0.51%) (Figure 4A). Next, we tested whether the wild type and the variant epitope were recognized by the same or different T-cell population(s). This was possible by co-staining PBMCs with two separate multimers presenting the reference and variant epitope labeled with different fluorochromes. The results clearly showed that two different antigen-specific T-cell populations recognized the wild type and the variant *M. tb* epitope (Figure 4B).

### Biochemical factors influencing antigen-specific T-cell recognition

**Figure 5:**
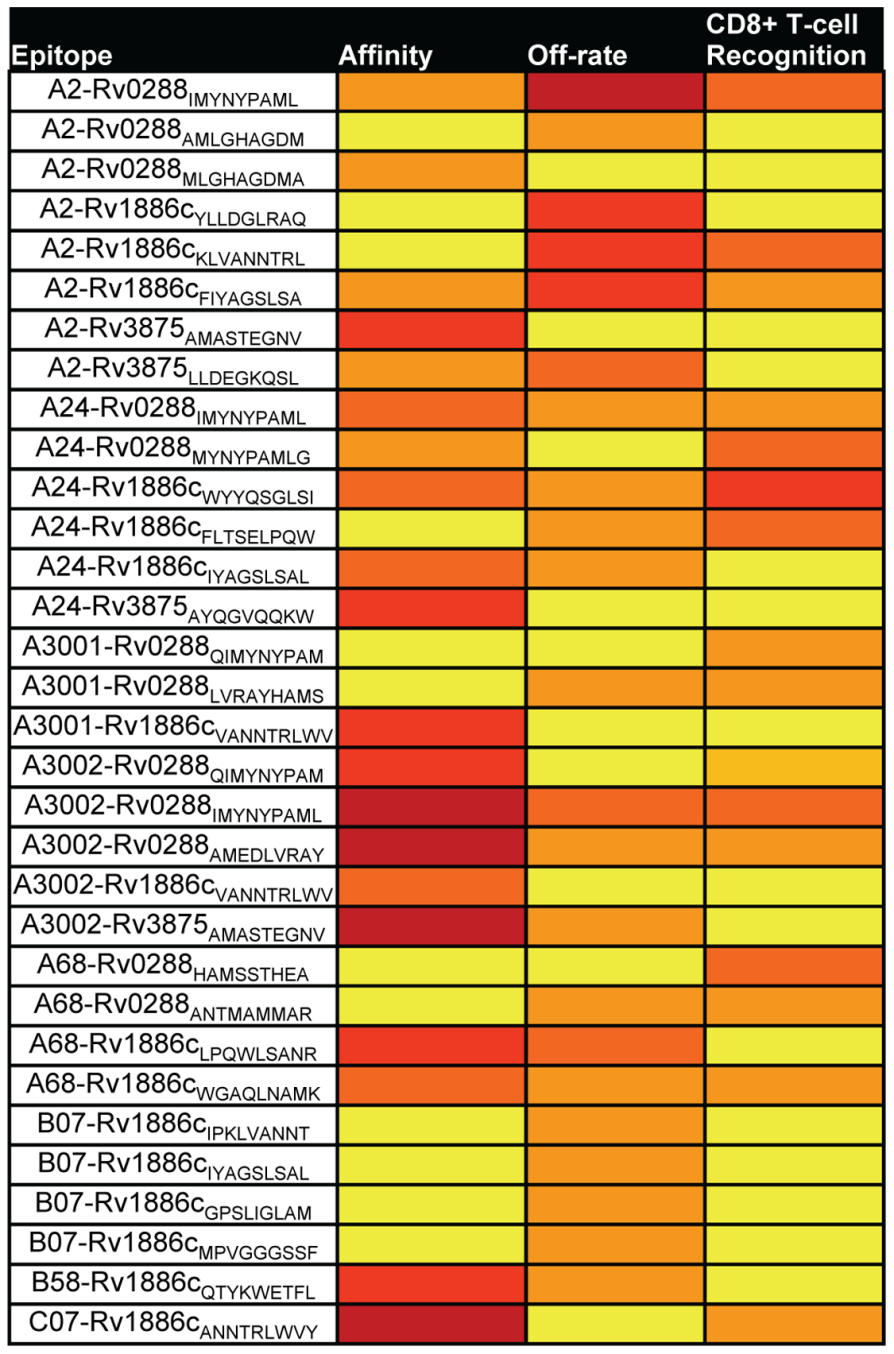
Heat-map comparing epitope-MHC affinity, epitope-MHC dissociation rate and antigen-specific CD8+ T-cell recognition. The affinity was divided into the following groups <10 nM (dark red), 10–70 nM (light red), 70–500 nM (orange), 500–1000 nM (dark yellow) and >1 µM (light yellow). The dissociation-rate (off-rate) were divided according to >10 h (dark red), 6–10 h (light red), 3–6 h (orange), 1.5–3 h (dark yellow) and <1.5 h (light yellow). Finally, the T-cell recognition was grouped as follows; >1% antigen-specific CD8+ T-cells recognizing the epitope (dark red), 0.6–1% (light red), 0.4–0.6% (orange), 0.2–0.4% (dark yellow) and <0.2% (light yellow).

Previous data suggested correlations between high MHC class I-peptide affinity and dominant T-cell responses [46]. We compared therefore the binding characteristics of the p-MHC complex with the actual frequency of antigen-specific T-cells recognizing a specific epitope. Based on our data concerning affinity, dissociation rate and *M. tb* epitope-specific recognition frequencies, it was possible to determine that among the 7 epitopes, which showed the highest average antigen-specific recognition (>0.4%), the affinity varied considerably (between 6 µM and 8 nM), as well as the off-rate (0.5 h–27 h). In addition, we could detect allele-specific characteristics such as very high affinity regarding all the HLA-A*30 restricted epitopes and the opposite was found to be true regarding the B*07 restricted epitopes. However, this did not correlate with the frequency of antigen-specific CD8+ T-cells (in PBMCs from patients with active TB). The highest frequency of *M. tb*-specific T-cells for almost all epitopes was restricted by HLA-A*24 (Figure 5).

## Discussion

Our study shows that, at all three levels (MHC binding, T-cell-recognition and the nature of the specific T-cells), we were able to detect allele-specific differences which potentially affect CD8+ T-cell immune-recognition. Regarding the p-MHC associations, we were able to confirm a broad peptide-binding repertoire for some alleles (e.g. HLA-A*02:01, A*24:02 and A*30:02) and a more restricted peptide-binding repertoire for other alleles (e.g. A*30:01 and B*07:02). This is in line with previously reported data (yet with different peptide ligands) [17], [24], [39], [47]. In addition, we included the less well studied alleles A*68:01 and B*58:01 as well as the (not yet reported) allele C*07:01. A*68:01 and B*58:01 showed a relatively broad binding repertoire, while C*07:01 showed a very restricted peptide binding. Structural constrains, electrostatic charge and hydrophobicity within the MHC binding pockets dictate the binding peptide repertoire [48] and are therefore part of the explanation why inter-allelic differences can be seen in the broadness of the peptide binding repertoires. In addition, these factors influence the stability of the p-MHC complex and might therefore explain some of the inter-allelic differences that could be seen in affinity and peptide dissociation rates.

The stability and off-rate are factors that are biologically important; they determine the time frame in which a certain epitope would be available for T-cell priming and for recognition in the effector phase of the immune response. The allele-specific differences in the p-MHC dissociation rate probably originate from intrinsic structural features of the allotype. This would imply that not only a limited number of epitopes would be available for presentation by these alleles but also that the time-frame in which the presentation could occur would be shorter. This scenario might entail a disadvantage for immune-recognition in individuals with certain allotypes, yet a fast peptide off-rate seems to be able to be counter-balanced by a high affinity, as shown for a number of *M. tb* epitopes included in this study. Affinities (ED50) between 100 µM and 10 nM have previously been identified using a similar experimental design and our findings in this study are in line with these data [24], [39].

We identified highly promiscuous ‘hot-spots’ in *M. tb* proteins of inter-allelic binding such as Rv1886c [(KL)VANNTRL(WV) and (L)AAYHPQQF(I)] and Rv0288 [(QI)MYNYPAM(LG)], adding more alleles to the already identified hotspot in this protein [17], [24]. For some epitopes, promiscuous binding to up to 7 different HLA class I alleles including members of both HLA-A, B and C allele families have been identified in this study. In addition, some of these areas have previously been shown to be able to bind to the MHC class II alleles DR1, DR2 and DR4 [32]. We have also been able to show allele-independent T-cell recognition for the Rv0288 derived epitope between five different HLA-A alleles. Since the individual T-cell responses were found to be similar, concerning T-cell frequency, T-cell phenotype and effector functions, these immunological ‘hot-spots’ might be potential candidates for peptide-based vaccine-strategies since the allelic coverage would include a large part of the world population, including the most common HLA-A allele in Caucasians (A*02), in Asians (A*24) and in Africans (A*30) [9].

Even though the importance of different MHC class I allotypes in shaping the possible peptide-repertoires is well established, we know in fact very little about how inter-allelic variations affect the generation, phenotype and functionality of a pathogen-specific T-cell repertoire. The immune-recognition by antigen-specific CD8+ T-cells in many diseases is often directed against a few of the many potential epitopes originating from a complex pathogen. This ‘skewing’ of the immune system gives rise to immune-dominant epitopes, which might be accompanied by less recognized sub-dominant epitopes [49]. The driving force behind immune-dominance and sub-dominance in different disease settings is not completely understood, but availability (time and receptor numbers) of a certain p-MHC complex on the cell-surface [50], as well as conformation of the epitope presented [51] and frequencies of T-cells precursors with the possibility of recognizing the epitope [52] seem to be crucial factors in this process. In this study, we were able to detect a dominant response in blood from some patients, for instance against the A*24 restricted Rv3875 epitope ELNNALQNL. This is interesting, since the majority of previously described dominant *M. tb.* epitopes seem to be HLA-B restricted [24], [53]. However, T-cell immune-dominance concerning a single epitope seems to be quite rare in active *M. tb* infection, since the mapping using 48 different multimers (some were previously described [17], [24], [40], [41], [54]) has not revealed a single dominant CD8+ T-cell epitope, yet a low frequency of a broad panel of co-dominant *M. tb* epitopes in the peripheral circulation of most patients infected with *M. tb*. The dominance and the immunological profile might however differ between the site of infection and the peripheral blood cells used in this study, as previously reported [42].

The generation of a broader T-cell repertoire may protect from the emergence of escape mutations in viral disease. A broader T-cell repertoire targeting ‘subdominant’ epitope may not be associated with clinical efficacy in TB, since these samples were taken from patients who failed to contain their disease (all individuals in the current study exhibited active pulmonary disease). This seems to be more in line with the low frequency of disease-reactive T-cells in the peripheral circulation that previously has been seen in the context of cancer [55] or the loss of certain immune-dominant epitopes in the peripheral circulation in chronic infection with simian immunodeficiency virus (SIV). Future studies will show, whether dominant T-cell responses arise after effective TB treatment, whether such immune responses are associated with latent TB, which represents a broad clinical spectrum, or even with continuous exposure to *M. tb*.

Deciphering the phenotype of antigen-specific CD8+ T-cells is important in the context of memory formation as well as for immune effector functions [44], [45]. Previous studies have identified the majority of antigen-specific T-cells residing in memory (CD45RA−) [40], [56] or terminally differentiated (CD45RA+CCR7−) [41] T-cells. Yet, an increasing number of studies identified a majority of *M. tb*-specific or BCG-reactive T-cells in both adults and children belonging to ‘naïve’/precursor (CD45RA+CCR7+)/(CD45RA+CD28+) [17], [57], [58], [59] compartment. In this study, we were once again able to detect that the majority of *M. tb* antigen-specific T-cells resides in the (CD45RA+CCR7+) precursor population – in contrast to the phenotype of total CD8+ T-cells which showed equal number of naïve, effector memory and terminally differentiated cells. It has previously been speculated for HIV infection, where this phenomenon also has been observed, that the antigen-specific T-cells with cell-surface expression of both CD45RA and CCR7 might represent effector memory cells that reverted back to a ‘naïve’ like phenotype without losing this subset's ability of proliferation upon antigen-stimulation [45], yet it may also be due to increased lymphopoiesis in patients with active TB.

We detected allele-specific variations, rather than antigen-specific, regarding frequencies and the differentiation/maturation profile (based on CD45RA/CCR7) of antigen-specific T-cells. First, we hypothesized that this finding was due to differences in the presented *M. tb* epitope and thus due to the nature of the target protein, since the promiscuous epitopes were similarly recognized by inter-allelic restricted T-cells. However, when we compared several different epitopes, restricted by the same allele, allele-specific patterns of immune-recognition emerged while the antigen-specific patterns were remarkably constant. This was in contrast to the levels of vaccine-induced antigen-specific T-cells which were found to be higher targeting epitopes originating from Rv1886c (compared to Rv0288) in one previous study [25], it was also in contrast to the different phenotype of antigen-specific cells found in another study [41]. However, the immune-profile of active TB disease and the vaccine-induced profile might not correlate, and in the second study the phenotypic profile of *only a single* epitope/protein was compared.

To our knowledge, no correlations between the T-cell profile and the restricting allele have been shown before in TB. One example has been reported in HIV infection where some alleles like B*57 and B*58, that have been associated with ‘elite controllers’, associated with higher numbers of specific cytokine producing cells [60]. Interestingly, the prevalent African alleles HLA-A*30 and C*07 restricted epitopes give rise to higher proportions of terminally differentiated effector CD8+ T-cells in African TB patients and this T-cell phenotype has been shown to be important in controlling the disease [3]. One possible explanation is that the long-standing co-evolution between mycobacteria and humans has taken part in the regional selection of the most prevalent MHC class I alleles based on an advantage in CD8+ T-cell responses, i.e. to expand immune effector T-cells. In line with this observation is also the fact that these alleles seem to restrict *M. tb* antigen specific T-cells with high CD107a expression *ex vivo* (indicating cytotoxic function).

We were able to detect biologically relevant differences concerning the naturally occurring amino-acid substitutions in the protein Rv0288. Since *M. tb* previously has been shown to express low sequence variability in most of its epitopes, it was speculated that some immune-recognition might be beneficial for the bacteria, for instance in the establishment of a latent infection or by contributing to transmission [23]. We have studied the effect of the epitope-based polymorphism on both the MHC level as well as on the TCR recognition level for the protein Rv0288. An explanation for the higher frequency of amino acid substitutions in this protein [23] (indicating immune-evasion) might be that most of the substitutions are part of immunological hot-spots with broad inter-allelic immune recognition [17], [24]. We could show in the current study that most of the substitutions affected MHC class I binding negatively, indicating a potential loss of peptide presentation. Some of the substitutions reside within an anchor residue of the peptides and might therefore directly interfere with allele-specific peptide-binding. On the other hand, explanations for the negative effect of other substitutions might be that they cause steric hindrance, or changes in intra-peptide constrains changing the conformation of upward pointing residues [61]. The outcome of individual p-MHC-TCRs interactions is determined by affinity and avidity dictating positive and negative downstream signaling events such as CD3 zeta-chain phosphorylation [62]. In this study, we could still detect an epitope-specific antigen-specific T-cell population after introducing the amino acid substitution, although these T-cells were found to belong to a complete separate cell population as compared to the T-cells recognizing the original epitope. This change in TCR-repertoire usage upon amino acid substitution in an epitope has previously been seen in SIV infections [63] as well as within an influenza epitope restricted by H2-D^b^ [61]. It would be of interest to elucidate if these different T-cell populations exhibit similar functionality showing a full or partial agonistic, or even antagonistic, effect on the TCR repertoire recognizing wild-type or variant *M. tb* epitopes.

In conclusion, we constructed a broad panel of novel tools to be able to study *M. tb-*specific responses in African with TB infection. We could detect a low frequency of a broad repertoire of co-dominant *M. tb* epitopes in the peripheral circulation of patients with active TB. The study of host-pathogen effects on immune-recognition in *M. tb* derived CD8+ T-cell epitopes revealed 1) MHC class I allele specific differences in the broadness of the peptide-binding repertoire, 2) associations of the frequency and phenotype of *M. tb* antigen-specific T-cells with the MHC class I restricting alleles and 3) differences between variant *M. tb* epitopes concerning peptide binding and T-cell recognition. This study provides a first view of the nature of the anti-*M. tb* specific CD8+ response in patients with an African background. It is our hope that these findings, as well as these novel tools will aid future research on *M. tb* in Africa.

## Supporting Information

Figure S1 **Example of staining using the cell surface markers CD3, CD4, CD8, multimer, CD45RA, CCR7, CD107a and CD12 (PAT 1284 and PAT 3282) in African TB patients.** Numbers show the frequencies of cell positive for the specific marker/markers. The gating strategy was the following: Based on forward and side-scatter, lymphocytes were detected. From the lymphocytes, T-cells were enumerated using the CD3 marker. These cells were then divided into CD4+ T-cells and CD8+ T-cells based on expression of CD4 and CD8. In the CD8+ T-cells, multimer-specific cells as well as cells expressing the cell surface markers CD45RA, CCR7, CD107a and CD127 were detected. Finally, in the multimer positive cells the frequencies of cells expressing the markers CD45RA, CCR7, CD107a and CD127 were detected as well.
(TIF)

Figure S2 **Examples of positive and negative multimer staining.** The multimer specific cells were detected in the CD3+CD8+ population using negative multimers and epitope specific multimers presenting the following epitopes: FIYAGSLSA (HLA-A*02:01), ELNNALQNL (A*24:02), QIMYNYPAM (A*30:01 and A*30:02), HAMSSTHEA (A*68:01), IPKLVANNT (B*07:02), QTYKWETFL (B*58:01) and ANNTRLWVY (C*07:01). Numbers are indicating frequencies of multimer-specific cells.
(TIF)

Figure S3 The antigen-specific recognition of the HLA-A*02:01 (A) and A*30 (A*30:01 – open symbols and A*30:02 – closed symbols) (B) restricted multimers, each dot represents the individual staining in one patient, the colors represents the M. tb protein and the derivative epitope (Rv0288 – black, Rv1886c – red, Rv3875 – blue and Rv2958c, Rv2957 and Rv0447c – green).
(TIF)

Figure S4 Frequency of total CD8+ and antigen-specific T-cells belonging to the (A) naïve compartment (CD45RA+, CCR7+) and (B) central memory compartment (CD45RA−, CCR7+) divided per restricting MHC class I allele (Total CD8+ T-cells – filled circles, A*02:01 – filled squares, A*24:02 – filled triangles, A*30:01/A*30:02 – filled diamonds, A*68:01 – open circles, B*07:02 – open squares, B*58:01 – open triangles and C*07:01 – open diamonds). Total CD8+ and antigen-specific T-cells belonging to the (C) naïve compartment (CD45RA+, CCR7+), central memory compartment (D) (CD45RA−, CCR7+), (E) effector memory compartment (CD45RA−, CCR7−) and (F) terminally differentiated compartment (CD45RA+, CCR7−) divided per immunogenic TB protein (Total CD8+ T-cells – circles, Rv0288 – squares, Rv1886c – triangles, Rv3875 – diamonds and antigens expressed primarily on slow growing bacteria (Rv2958c, Rv2957 and Rv0447c -stars). Each dot represents an individual tetramer in one individual TB patient.
(TIF)

Figure S5 Frequency of total CD8+ T-cell belonging to the (A) naïve compartment (CD45RA+, CCR7+), (B) central memory compartment (CD45RA−, CCR7+), (C) effector memory compartment (CD45RA−, CCR7−) and (D) terminally differentiated compartment (CD45RA+, CCR7−) specific for the ‘super-epitope’ (QI)MYNYPAM(LG) (A*02:01-IMYNYPAML – black circles, A*24:02-IMYNYPAML – dark grey circles, A*24:02-MYNYPAMLG – dark grey squares, A*30:01-QIMYNYPAM – light grey triangles, A*30:02-QIMYNYPAM – open triangles and A*30:02-IMYNYPAML – open circles). Each dot represents an individual tetramer in one individual TB patient.
(TIF)

Figure S6 **Frequency of total CD8+ and antigen-specific T-cells expressing (A) CD107a and (B) CD127 divided per epitope derived protein (Total CD8+ T-cells – circles, Rv0288 – squares, Rv1886c – triangles, Rv3875 – diamonds) and antigens expressed primarily on slow growing bacteria (Rv2958c, Rv2957 and Rv0447c – stars).** CD8+ T-cells expressing (C) CD107a and (D) CD127 specific for the ‘super-epitope’ (QI)MYNYPAM(LG) (A*02:01-IMYNYPAML – black circles, A*24:02-IMYNYPAML – dark grey circles, A*24:02-MYNYPAMLG – dark grey squares, A*30:01-QIMYNYPAM – light grey triangles, A*30:02-QIMYNYPAM – open triangles and A*30:02-IMYNYPAML – open circles). Each dot represents an individual tetramer in one individual TB patient. Each dot represents an individual tetramer in one individual TB patient.
(TIF)

Table S1 **Demographic data of the included patients.**
(PDF)

Table S2 **MHC class I binding affinity and off-rate data for peptide-epitopes derived from Rv1886c (Ag85B).**
(PDF)

Table S3 **Frequency of epitope-specific T cells identified by multimer staining.**
(PDF)
