# Supplementary figures and images for: A Broad Profile of Co-Dominant Epitopes Shapes the Peripheral Mycobacterium tuberculosis Specific CD8+ T-Cell Immune Response in South African Patients with Active Tuberculosis
Source: PLoS One. 2013 Mar 26;8(3):e58309. doi: 10.1371/journal.pone.0058309 (PMC3608651; doi:10.1371/journal.pone.0058309)

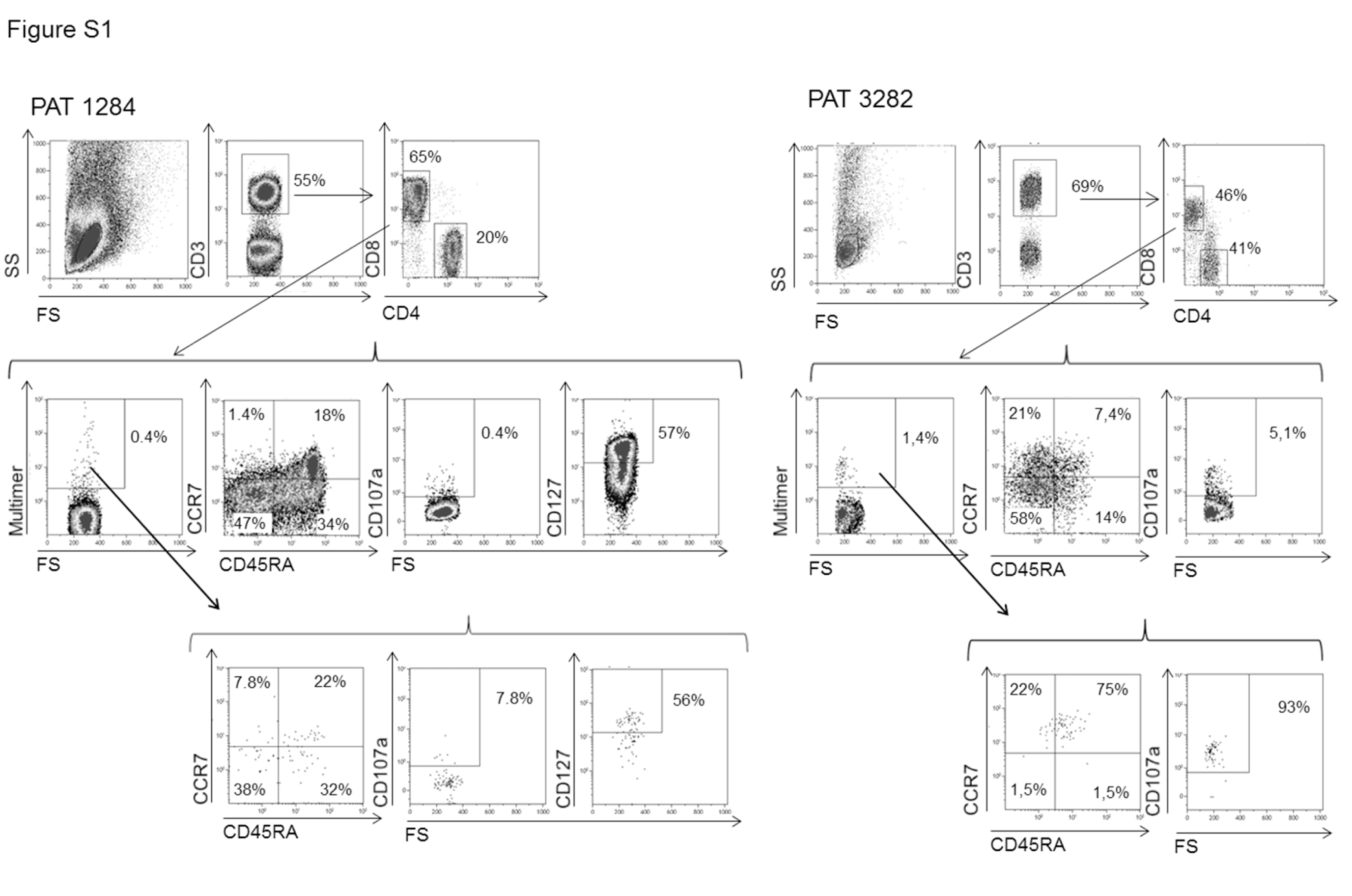

Supplement: Figure S1 — Example of staining using the cell surface markers CD3, CD4, CD8, multimer, CD45RA, CCR7, CD107a and CD12 (PAT 1284 and PAT 3282) in African TB patients. Numbers show the frequencies of cell positive for the specific marker/markers. The gating strategy was the following: Based on forward and side-scatter, lymphocytes were detected. From the lymphocytes, T-cells were enumerated using the CD3 marker. These cells were then divided into CD4+ T-cells and CD8+ T-cells based on expression of CD4 and CD8. In the CD8+ T-cells, multimer-specific cells as well as cells expressing the cell surface markers CD45RA, CCR7, CD107a and CD127 were detected. Finally, in the multimer positive cells the frequencies of cells expressing the markers CD45RA, CCR7, CD107a and CD127 were detected as well. (TIF) [file pone.0058309.s001.tif]

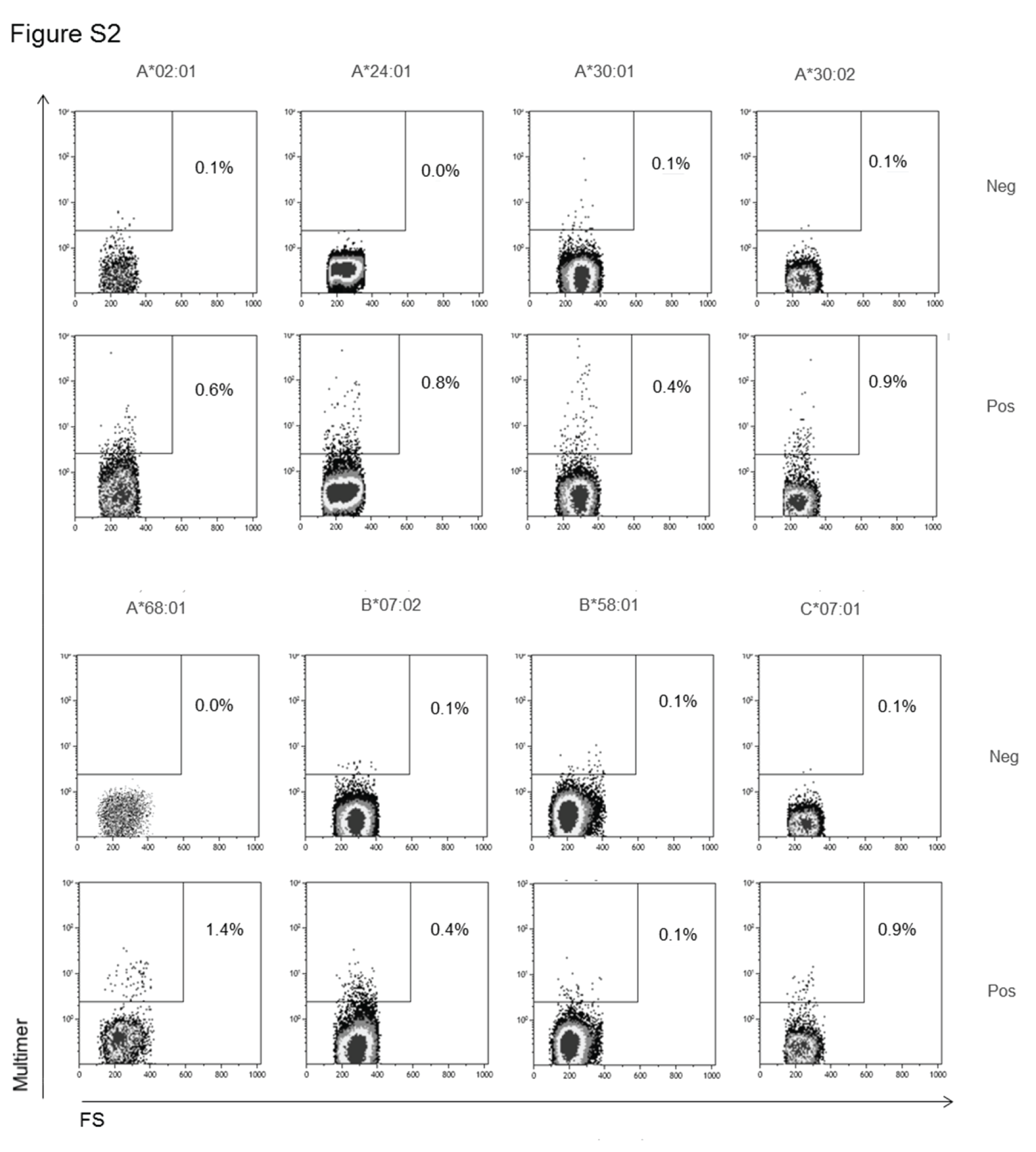

Supplement: Figure S2 — Examples of positive and negative multimer staining. The multimer specific cells were detected in the CD3+CD8+ population using negative multimers and epitope specific multimers presenting the following epitopes: FIYAGSLSA (HLA-A*02:01), ELNNALQNL (A*24:02), QIMYNYPAM (A*30:01 and A*30:02), HAMSSTHEA (A*68:01), IPKLVANNT (B*07:02), QTYKWETFL (B*58:01) and ANNTRLWVY (C*07:01). Numbers are indicating frequencies of multimer-specific cells. (TIF) [file pone.0058309.s002.tif]

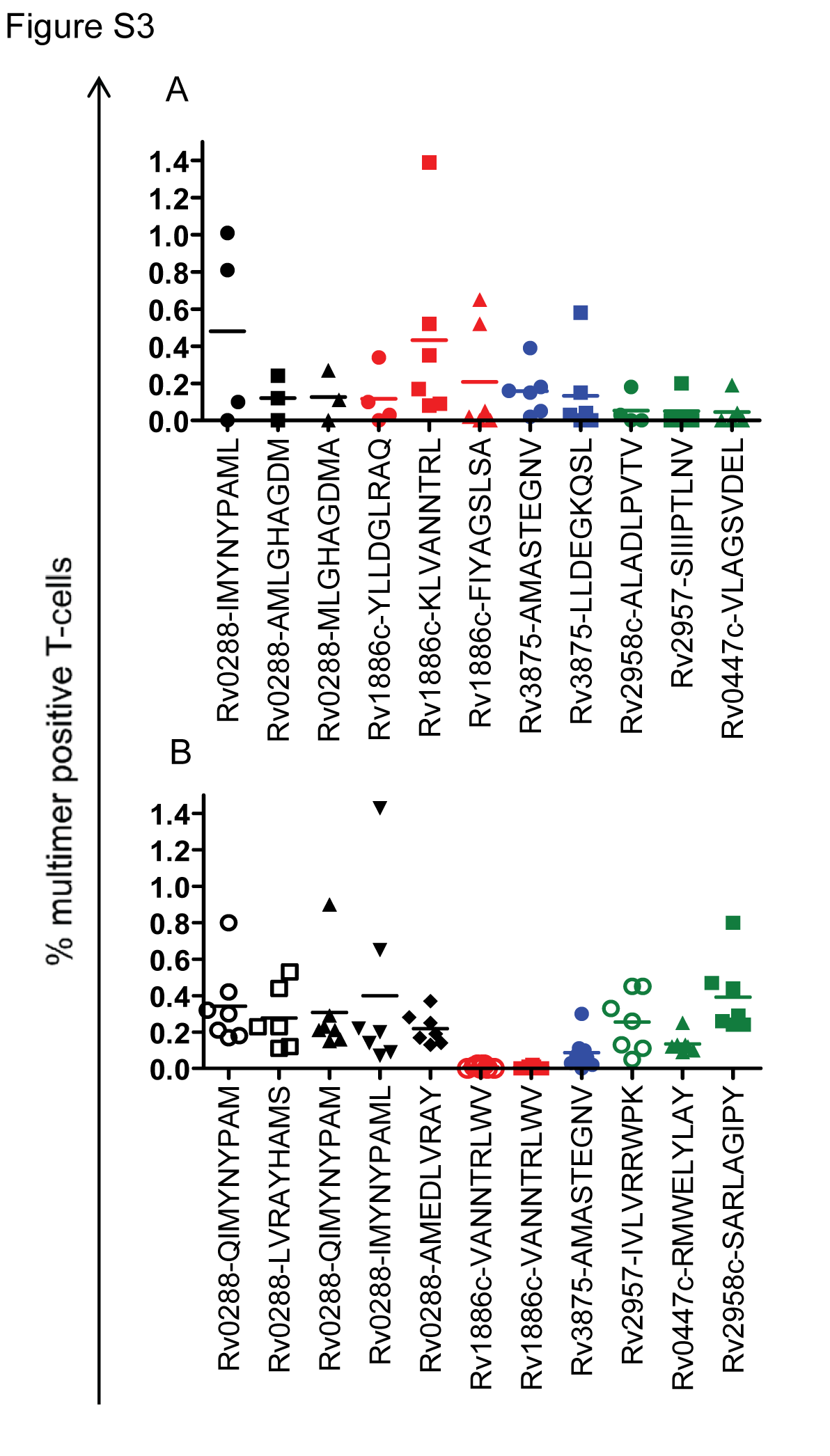

Supplement: Figure S3 — The antigen-specific recognition of the HLA-A*02:01 (A) and A*30 (A*30:01 – open symbols and A*30:02 – closed symbols) (B) restricted multimers, each dot represents the individual staining in one patient, the colors represents the M. tb protein and the derivative epitope (Rv0288 – black, Rv1886c – red, Rv3875 – blue and Rv2958c, Rv2957 and Rv0447c – green). (TIF) [file pone.0058309.s003.tif]

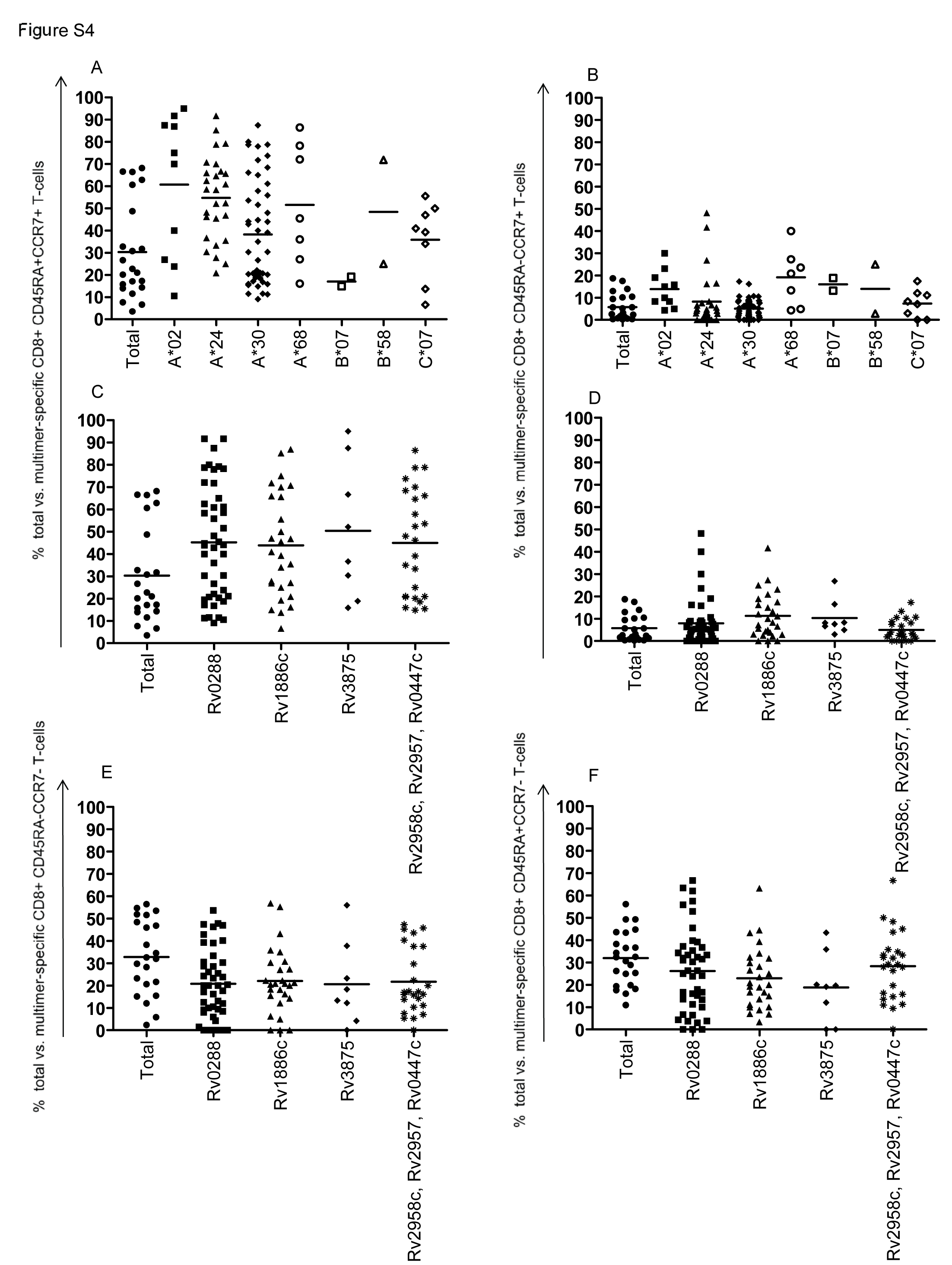

Supplement: Figure S4 — Frequency of total CD8+ and antigen-specific T-cells belonging to the (A) naïve compartment (CD45RA+, CCR7+) and (B) central memory compartment (CD45RA−, CCR7+) divided per restricting MHC class I allele (Total CD8+ T-cells – filled circles, A*02:01 – filled squares, A*24:02 – filled triangles, A*30:01/A*30:02 – filled diamonds, A*68:01 – open circles, B*07:02 – open squares, B*58:01 – open triangles and C*07:01 – open diamonds). Total CD8+ and antigen-specific T-cells belonging to the (C) naïve compartment (CD45RA+, CCR7+), central memory compartment (D) (CD45RA−, CCR7+), (E) effector memory compartment (CD45RA−, CCR7−) and (F) terminally differentiated compartment (CD45RA+, CCR7−) divided per immunogenic TB protein (Total CD8+ T-cells – circles, Rv0288 – squares, Rv1886c – triangles, Rv3875 – diamonds and antigens expressed primarily on slow growing bacteria (Rv2958c, Rv2957 and Rv0447c -stars). Each dot represents an individual tetramer in one individual TB patient. (TIF) [file pone.0058309.s004.tif]

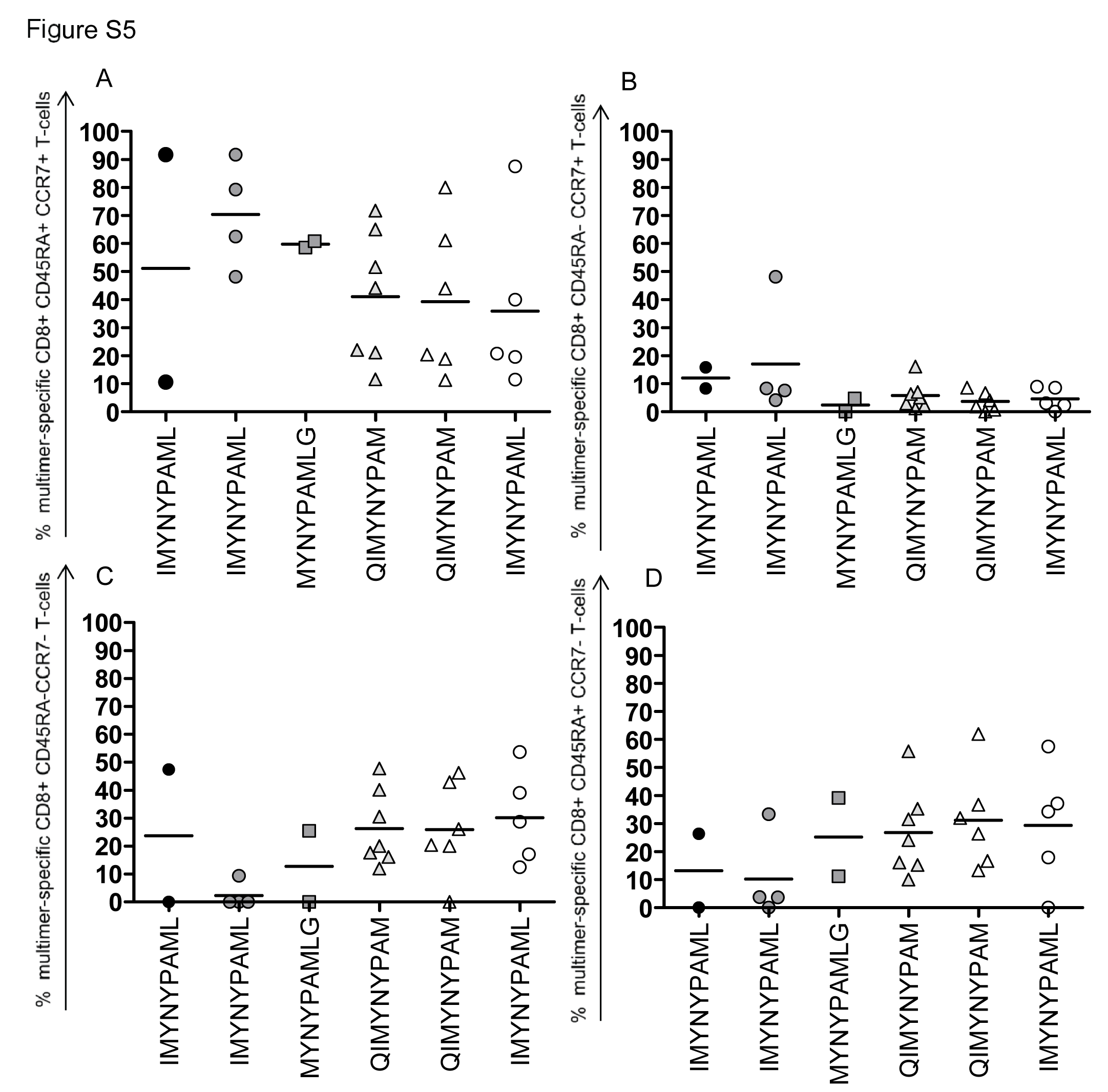

Supplement: Figure S5 — Frequency of total CD8+ T-cell belonging to the (A) naïve compartment (CD45RA+, CCR7+), (B) central memory compartment (CD45RA−, CCR7+), (C) effector memory compartment (CD45RA−, CCR7−) and (D) terminally differentiated compartment (CD45RA+, CCR7−) specific for the ‘super-epitope’ (QI)MYNYPAM(LG) (A*02:01-IMYNYPAML – black circles, A*24:02-IMYNYPAML – dark grey circles, A*24:02-MYNYPAMLG – dark grey squares, A*30:01-QIMYNYPAM – light grey triangles, A*30:02-QIMYNYPAM – open triangles and A*30:02-IMYNYPAML – open circles). Each dot represents an individual tetramer in one individual TB patient. (TIF) [file pone.0058309.s005.tif]

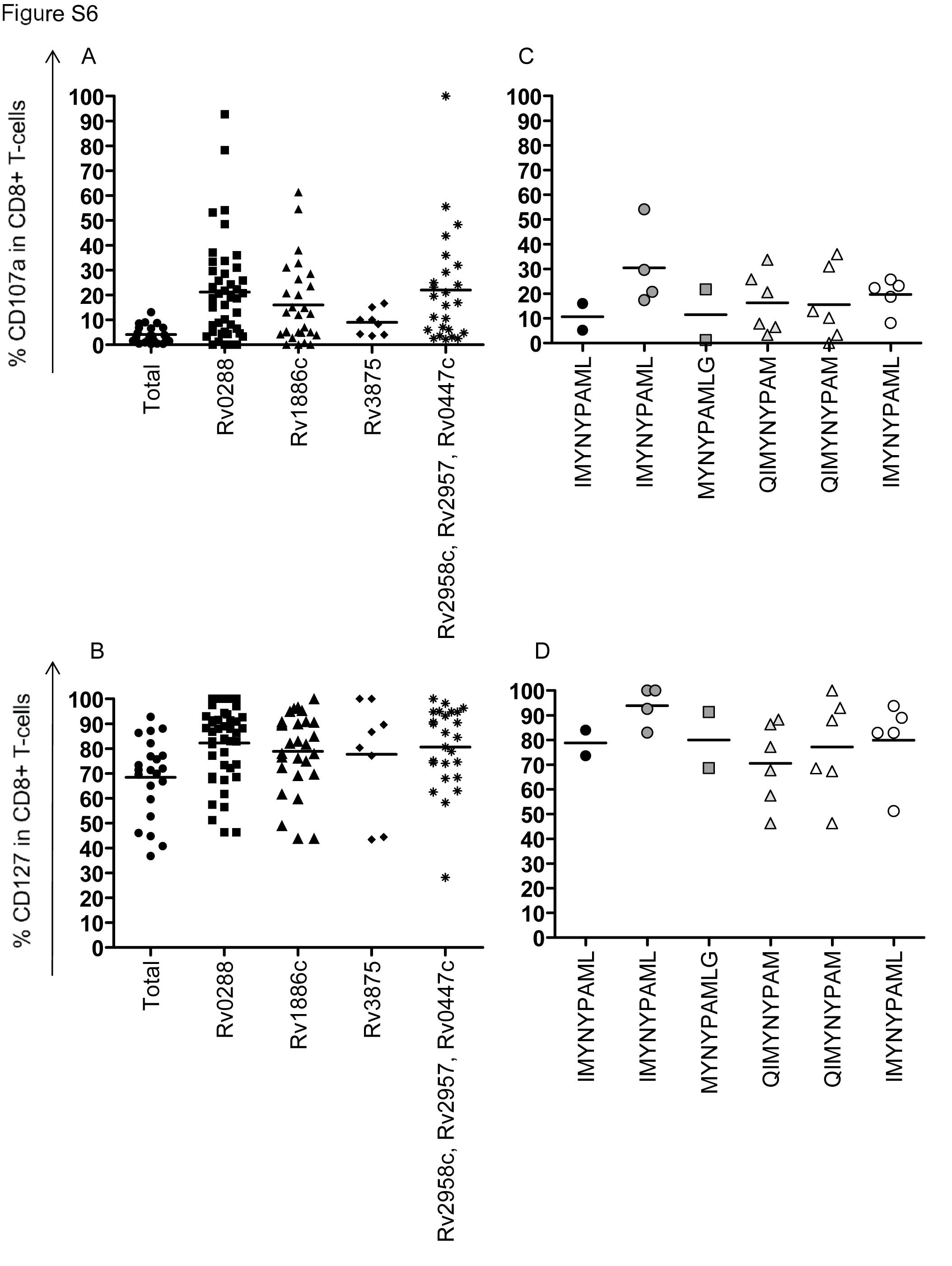

Supplement: Figure S6 — Frequency of total CD8+ and antigen-specific T-cells expressing (A) CD107a and (B) CD127 divided per epitope derived protein (Total CD8+ T-cells – circles, Rv0288 – squares, Rv1886c – triangles, Rv3875 – diamonds) and antigens expressed primarily on slow growing bacteria (Rv2958c, Rv2957 and Rv0447c – stars). CD8+ T-cells expressing (C) CD107a and (D) CD127 specific for the ‘super-epitope’ (QI)MYNYPAM(LG) (A*02:01-IMYNYPAML – black circles, A*24:02-IMYNYPAML – dark grey circles, A*24:02-MYNYPAMLG – dark grey squares, A*30:01-QIMYNYPAM – light grey triangles, A*30:02-QIMYNYPAM – open triangles and A*30:02-IMYNYPAML – open circles). Each dot represents an individual tetramer in one individual TB patient. Each dot represents an individual tetramer in one individual TB patient. (TIF) [file pone.0058309.s006.tif]
