# Supplementary material for: A Broad Profile of Co-Dominant Epitopes Shapes the Peripheral Mycobacterium tuberculosis Specific CD8+ T-Cell Immune Response in South African Patients with Active Tuberculosis
Source: PLoS One. 2013 Mar 26;8(3):e58309. doi: 10.1371/journal.pone.0058309 (PMC3608651; doi:10.1371/journal.pone.0058309)
Supplement: Table S1 — Demographic data of the included patients. (PDF) [file pone.0058309.s007.pdf]

**Table S1.** Demographic patient data

| ID | Sex | Age | Ethnicity |
|---|---|---|---|
| 1265 | F | 45 | Black/Colored |
| 1284 | F | 46 | Black/Colored |
| 1288 | M | 44 | Black/Colored |
| 1292 | M | 45 | Black/Colored |
| 1421 | M | 40 | Black/Colored |
| 1903 | F | 36 | Black/Colored |
| 3104 | F | 31 | Black/Colored |
| 3112 | F | 55 | Black/Colored |
| 3245 | M | 32 | Black/Colored |
| 3246 | M | 26 | Black/Colored |
| 3282 | M | 59 | Black/Colored |
| 3283 | F | 33 | Black/Colored |
| 4063 | n.d. | n.d. | Black/Colored |
| 5363 | F | 43 | Black/Colored |
| 5364 | M | 30 | Black/Colored |
| 5436 | F | 20 | Black/Colored |
| 5437 | F | 22 | Black/Colored |
| 5562 | F | 31 | Black/Colored |
| 5905 | F | 17 | Black/Colored |
| 6088 | F | 16 | Black/Colored |
| 6089 | M | 25 | Black/Colored |
| 7235 | F | 20 | Black/Colored |
| 7237 | F | 32 | Black/Colored |
| 7238 | F | 29 | Black/Colored |
| 7606 | F | 48 | Black/Colored |
| 7767 | F | 35 | Black/Colored |
| 7769 | F | 26 | Black/Colored |
