# Supplementary material for: A Broad Profile of Co-Dominant Epitopes Shapes the Peripheral Mycobacterium tuberculosis Specific CD8+ T-Cell Immune Response in South African Patients with Active Tuberculosis
Source: PLoS One. 2013 Mar 26;8(3):e58309. doi: 10.1371/journal.pone.0058309 (PMC3608651; doi:10.1371/journal.pone.0058309)
Supplement: Table S2 — MHC class I binding affinity and off-rate data for peptide-epitopes derived from Rv1886c (Ag85B). (PDF) [file pone.0058309.s008.pdf]

Table S2. MHC class I binding affinity and off-rate data for peptide-epitopes derived from Rv1886c (Ag85B).

		A*02:01			A*24:02			A*30:01			A*30:02			A*68:01			B*07:02			B*58:01			C*07:01		
**Peptide ID**	**Sequence***	**Bind****	**Aff**	**O-rate**	**Bind**	**Aff**	**O-rate**	**Bind**	**Aff**	**O-rate**	**Bind**	**Aff**	**O-rate**	**Bind**	**Aff**	**O-rate**	**Bind**	**Aff**	**O-rate**	**Bind**	**Aff**	**O-rate**	**Bind**	**Aff**	**O-rate**
Rv1886c$_{58-66}$	**MGRDIKVQF**	0			25			0			**61**	7E-08	0.3	10			**41**	8E-07	0.3	**61**	3E-07	0.3	**68**	8E-08	0.2
Rv1886c$_{64-72}$	VQFQSGGNN	0			0			0			**51**	n.d.	n.d.	0			0			0			0		
Rv1886c$_{69-77}$	*GGNNSPAVY*	0			0			0			**90**	n.d.	n.d.	3			0			0			**45**	n.d.	n.d.
Rv1886c$_{70-78}$	GNNSPAVYL	0			6			0			0			3			0			0			**51**	n.d.	n.d.
Rv1886c$_{71-79}$	*NNSPAVYLL*	16			**49**	n.d.	n.d.	0			0			28			0			0			**70**	2E-07	0.6
Rv1886c$_{73-81}$	SPAVYLLDG	0			**49**	n.d.	n.d.	0			0			0			0			0			1		
Rv1886c$_{74-82}$	*PAVYLLDGL*	9			38			0			**120**	9E-08	1	2			**63**	n.d.	n.d.	0			17		
Rv1886c$_{75-83}$	*AVYLLDGLR*	24			8			1			**115**	2E-07	1.2	**114**	2E-07	1.1	0			0			4		
Rv1886c$_{76-84}$	**VYLLDGLRA**	**86**	n.d.	n.d.	**87**	n.d.	n.d.	0			**98**	n.d.	n.d.	13			0			0			9		
Rv1886c$_{77-85}$	YLLDGLRAQ	**111**	n.d.	n.d.	0			0			10			15			0			0			0		
Rv1886c$_{78-86}$	*LLDGLRAQD*	**63**	n.d.	n.d.	0			8			**140**	n.d.	n.d.	16			0			0			0		
Rv1886c$_{80-88}$	DGLRAQDDY	0			0			2			**58**	n.d.	n.d.	4			1			0			0		
Rv1886c$_{83-91}$	**RAQDDYNGW**	0			**65**	n.d.	n.d.	11			**119**	5E-07	1.2	2			0			**41**	n.d.	n.d.	2		
Rv1886c$_{89-97}$	NGWDINTPA	**87**	n.d.	n.d.	36			8			0			4			21			0			14		
Rv1886c$_{90-98}$	GWDINTPAF	0			**146**	1E-07	1.1	11			0			5			22			0			10		
Rv1886c$_{92-100}$	*DINTPAFEW*	0			**56**	n.d.	n.d.	7			0			3			9			**58**	n.d.	n.d.	10		
Rv1886c$_{93-101}$	**INTPAFEWY**	0			20			10			**132**	7E-07	1.9	9			2			**54**	5E-07	0.5	**41**	6E-07	1
Rv1886c$_{94-102}$	**NTPAFEWYY**	0			0			6			**99**	2E-08	1.5	**69**	1E-07	0.2	0			36			**48**	8E-09	0.9
Rv1886c$_{96-104}$	PAFEWYYQS	**76**	n.d.	n.d.	24			0			0			6			15			0			2		
Rv1886c$_{98-106}$	FEWYYQSGL	**124**	7E-07	0.8	37			10			26			10			11			0			15		
Rv1886c$_{100-108}$	WYYQSGLSI	37			**143**	n.d.	n.d.	18			0			0			6			0			13		
Rv1886c$_{101-109}$	*YYQSGLSIV*	**98**	n.d.	n.d.	**130**	n.d.	n.d.	29			37			5			0			0			25		
Rv1886c$_{102-110}$	**YQSGLSIVM**	**142**	5E-08	3.1	**59**	5E-07	1.5	27			**123**	8E-07	0.4	7			2			0			**68**	7E-09	0.3
Rv1886c$_{103-111}$	QSGLSIVMP	30			0			34			0			0			0			0			**43**	n.d.	n.d.
Rv1886c$_{104-112}$	SGLSIVMPV	**95**	n.d	n.d.	0			0			38			0			0			0			0		
Rv1886c$_{105-113}$	GLSIVMPVG	**100**	7E-08	2.1	0			0			0			0			0			0			0		
Rv1886c$_{106-114}$	LSIVMPVGG	33			0			0			**46**	n.d.	n.d.	0			0			29			0		
Rv1886c$_{108-116}$	**IVMPVGGQS**	**44**	n.d.	n.d.	0			0			**56**	n.d.	n.d.	0			**51**	n.d.	n.d.	0			0		
Rv1886c$_{109-117}$	VMPVGGQSS	**60**	n.d.	n.d.	0			0			12			0			0			0			0		
Rv1886c$_{110-118}$	MPVGGQSSF	0			0			0			0			0			**76**	n.d.	n.d.	0			0		
Rv1886c$_{111-119}$	PVGGQSSFY	0			0			0			**82**	n.d.	n.d.	0			0			0			0		
Rv1886c$_{114-122}$	**GQSSFYSDW**	27			**76**	n.d.	n.d.	0			**71**	2E-08	3.3	0			0			**62**	n.d.	n.d.	0		
Rv1886c$_{115-123}$	*QSSFYSDWY*	0			0			0			**90**	6E-07	2.2	**82**	1E-06	0.4	0			39			22		
Rv1886c$_{116-124}$	**SSFYSDWYS**	**53**	n.d.	n.d.	0			0			**72**	n.d.	n.d.	**103**	1E-07	0.1	0			0			1		
Rv1886c$_{117-125}$	SFYSDWYSP	7			0			0			**77**	n.d.	n.d.	0			0			0			0		
Rv1886c$_{118-126}$	FYSDWYSPA	**61**	n.d.	n.d.	10			13			0			0			0			0			0		
Rv1886c$_{119-127}$	*YSDWYSPAC*	**59**	n.d.	n.d.	0			15			2			18			0			**85**	n.d.	n.d.	5		
Rv1886c$_{121-129}$	DWYSPACGK	10			0			11			0			**136**	2E-07	0.6	0			0			0		
Rv1886c$_{124-132}$	SPACGKAGC	16			0			0			0			0			**77**	n.d.	n.d.	0			0		
Rv1886c$_{126-134}$	ACGKAGCQT	**44**	n.d.	n.d.	25			19			0			13			9			0			0		
Rv1886c$_{127-135}$	**CGKAGCQTY**	**44**	n.d.	n.d.	32			28			**138**	8E-08	3	**60**	n.d.	n.d.	8			35			5		
Rv1886c$_{128-136}$	*GKAGCQTYK*	33			19			34			**58**	n.d.	n.d.	**72**	n.d.	n.d.	9			22			0		
Rv1886c$_{129-137}$	**KAGCQTYKW**	22			**140**	7E-08	1.9	13			**45**	n.d.	n.d.	25			9			**193**	3E-08	8	31		
Rv1886c$_{130-138}$	AGCQTYKWE	12			31			3			18			4			2			**121**	2E-07	2	0		
Rv1886c$_{132-140}$	CQTYKWETF	2			**141**	3E-08	2.8	0			14			0			5			0			0		
Rv1886c$_{133-141}$	**QTYKWETFL**	**60**	3E-07	2.6	**110**	2E-08	2	0			**41**	2E-08	1	**46**	2E-07	0.7	0			**40**	2E-08	1.5	15		
Rv1886c$_{134-142}$	TYKWETFLT	36			**118**	2E-06	0.5	0			13			4			3			0			0		
Rv1886c$_{137-145}$	WETFLTSEL	14			18			12			1			**67**	n.d.	n.d.	15			29			3		

		A*02:01			A*24:02			A*30:01			A*30:02			A*68:01			B*07:02			B*58:01			C*07:01		
**Peptide ID**	**Sequence***	**Bind****	**Aff**	**O-rate**	**Bind**	**Aff**	**O-rate**	**Bind**	**Aff**	**O-rate**	**Bind**	**Aff**	**O-rate**	**Bind**	**Aff**	**O-rate**	**Bind**	**Aff**	**O-rate**	**Bind**	**Aff**	**O-rate**	**Bind**	**Aff**	**O-rate**
Rv1886c$_{138-146}$	ETFLTSELP	25			2			4			0			**103**	7E-07	0.1	5			16			0		
Rv1886c$_{140-146}$	**FLTSELPQW**	**75**	n.d.	n.d.	**117**	n.d.	n.d.	0			3			5			8			**71**	n.d.	n.d.	0		
Rv1886c$_{141-149}$	LTSELPQWL	**56**	n.d.	n.d.	9			0			19			0			7			34			2		
Rv1886c$_{142-150}$	TSELPQWLS	12			**52**	n.d.	n.d.	0			0			0			14			0			0		
Rv1886c$_{144-152}$	ELPQWLSAN	15			**56**	n.d.	n.d.	20			0			31			16			0			0		
Rv1886c$_{145-153}$	LPQWLSANR	6			26			29			0			**167**	6E-08	4.1	31			0			0		
Rv1886c$_{146-154}$	*PQWLSANRA*	**52**	n.d.	n.d.	**75**	n.d.	n.d.	36			13			13			14			0			2		
Rv1886c$_{147-155}$	*QWLSANRAV*	21			**110**	2E-06	0.4	39			**120**	7E-08	1.2	13			25			0			11		
Rv1886c$_{148-156}$	**WLSANRAVK**	13			**40**	n.d.	n.d.	**179**	1E-06	0.5	6			**139**	2E-07	2.1	**49**	2E-06	0.7	1			16		
Rv1886c$_{149-157}$	LSANRAVKP	17			27			**49**	n.d.	n.d.	15			0			11			0			24		
Rv1886c$_{153-161}$	**RAVKPTGSA**	19			0			4			18			10			**84**	n.d.	n.d.	**72**	5E-07	0.3	**82**	2E-07	1.4
Rv1886c$_{154-162}$	AVKPTGSAA	10			0			0			7			0			**82**	n.d.	n.d.	0			0		
Rv1886c$_{155-163}$	VKPTGSAAI	0			0			0			0			0			**43**	n.d.	n.d.	0			0		
Rv1886c$_{156-164}$	KPTGSAAIG	0			0			0			0			0			**51**	n.d.	n.d.	0			0		
Rv1886c$_{159-167}$	GSAAIGLSM	31			7			0			9			5			38			38			**66**	n.d.	n.d.
Rv1886c$_{160-168}$	SAAIGLSMA	**67**	n.d.	n.d.	0			0			0			0			31			5			0		
Rv1886c$_{164-172}$	GLSMAGSSA	**75**	n.d.	n.d.	0			0			0			0			31			0			0		
Rv1886c$_{165-173}$	**LSMAGSSAM**	**72**	2E-07	1.4	14			0			**79**	3E-06	1.1	**51**	1E-06	2.2	**92**	3E-07	2.6	0			**71**	3E-06	1.2
Rv1886c$_{166-174}$	**SMAGSSAMI**	**81**	n.d.	n.d.	**52**	2E-07	0.4	0			**45**	9E-08	1.3	0			14			**95**	2E-07	1.6	0		
Rv1886c$_{167-175}$	**MAGSSAMIL**	**54**	7E-07	0.4	26			0			13			0			**57**	n.d.	n.d.	**55**	9E-09	0.8	**94**	1E-08	0.5
Rv1886c$_{168-176}$	AGSSAMILA	**56**	n.d.	n.d.	0			0			0			2			10			12			14		
Rv1886c$_{169-177}$	GSSAMILAA	**66**	n.d.	n.d.	0			0			0			4			5			29			8		
Rv1886c$_{170-178}$	**SSAMILAAY**	0			36			0			**118**	7E-09	3.6	**101**	1E-07	1.8	4			**90**	2E-08	0.8	**41**	4E-07	1.6
Rv1886c$_{171-179}$	*SAMILAAYH*	9			0			0			**111**	4E-08	2.2	**69**	n.d.	n.d.	7			0			15		
Rv1886c$_{172-180}$	AMILAAYHP	34			0			0			33			0			1			**64**	n.d.	n.d.	1		
Rv1886c$_{173-181}$	*MILAAYHPQ*	**56**	n.d.	n.d.	0			0			**82**	n.d.	n.d.	0			0			11			4		
Rv1886c$_{174-182}$	*ILAAYHPQQ*	**57**	n.d.	n.d.	0			1			**45**	n.d.	n.d.	0			1			30			1		
Rv1886c$_{175-183}$	**LAAYHPQQF**	39			**52**	n.d.	n.d.	0			**66**	3E-07	1.1	37			**74**	n.d.	n.d.	13			**84**	1E-07	0.4
Rv1886c$_{176-184}$	**AAYHPQQFI**	**56**	6E-07	1.2	**54**	n.d.	n.d.	**83**	2E-07	0.5	34			**47**	3E-05	3.8	9			**64**	4E-08	0.8	0		
Rv1886c$_{177-185}$	**AYHPQQFIY**	13			**60**	n.d.	n.d.	6			**111**	1E-07	1.9	5			0			35			**43**	n.d.	n.d.
Rv1886c$_{178-186}$	YHPQQFIYA	**49**	n.d.	n.d.	0			3			0			0			0			9			15		
Rv1886c$_{181-189}$	*QQFIYAGSL*	**42**	n.d.	n.d.	0			0			**80**	n.d.	n.d.	0			0			8			27		
Rv1886c$_{183-191}$	*FIYAGSLSA*	**79**	n.d.	n.d.	11			0			21			38			**70**	n.d.	n.d.	2			35		
Rv1886c$_{184-192}$	**IYAGSLSAL**	**44**	n.d.	n.d.	**107**	n.d.	n.d.	0			**116**	4E-06	0.5	4			**83**	n.d.	n.d.	16			0		
Rv1886c$_{185-193}$	**YAGSLSALL**	**66**	n.d.	n.d.	**81**	1E-06	1.2	0			**64**	2E-07	0.9	13			**41**	1E-05	0.6	**58**	3E-08	0.7	19		
Rv1886c$_{188-196}$	SLSALLDPS	**57**	n.d.	n.d.	4			2			3			0			1			18			11		
Rv1886c$_{190-198}$	SALLDPSQG	0			0			12			**62**	n.d.	n.d.	0			3			8			9		
Rv1886c$_{191-199}$	*ALLDPSQGM*	**60**	n.d.	n.d.	0			5			**86**	n.d.	n.d.	0			17			1			22		
Rv1886c$_{193-201}$	*LDPSQGMGP*	**41**	n.d.	n.d.	12			32			0			0			**43**	n.d.	n.d.	12			18		
Rv1886c$_{194-202}$	DPSQGMGPS	30			**58**	n.d.	n.d.	21			29			0			0			12			19		
Rv1886c$_{195-203}$	PSQGMGPSL	39			**56**	n.d.	n.d.	21			16			0			8			0			29		
Rv1886c$_{196-204}$	*SQGMGPSLI*	**50**	n.d.	n.d.	**55**	n.d.	n.d.	11			0			0			2			11			22		
Rv1886c$_{198-206}$	**GMGPSLIGL**	**84**	n.d.	n.d.	**53**	n.d.	n.d.	16			**46**	n.d.	n.d.	0			5			9			35		
Rv1886c$_{199-207}$	*MGPSLIGLA*	**68**	n.d.	n.d.	0			15			0			0			5			**41**	n.d.	n.d.	14		
Rv1886c$_{200-208}$	GPSLIGLAM	11			0			0			0			6			**85**	n.d.	n.d.	18			0		
Rv1886c$_{202-210}$	*SLIGLAMGD*	**57**	n.d.	n.d.	0			0			**47**	n.d.	n.d.	0			3			0			0		
Rv1886c$_{203-211}$	LIGLAMGDA	**59**	n.d.	n.d.	0			0			5			0			0			0			0		
Rv1886c$_{206-214}$	*LAMGDAGGY*	10			0			0			**74**	n.d.	n.d.	**56**	n.d.	n.d.	2			39			0		
Rv1886c$_{207-215}$	*AMGDAGGYK*	31			0			28			**80**	7E-07	1.6	**94**	5E-07	3.2	8			0			0		
Rv1886c$_{208-216}$	MGDAGGYKA	**45**	n.d.	n.d.	0			0			0			9			0			0			0		
Rv1886c$_{211-219}$	AGGYKAADM	0			0			0			**85**	n.d.	n.d.	0			0			0			0		

Peptide ID	Sequence*	A*02:01			A*24:02			A*30:01			A*30:02			A*68:01			B*07:02			B*58:01			C*07:01		
		Bind**	Aff	O-rate	Bind	Aff	O-rate	Bind	Aff	O-rate	Bind	Aff	O-rate	Bind	Aff	O-rate	Bind	Aff	O-rate	Bind	Aff	O-rate	Bind	Aff	O-rate
Rv1886c$_{212-220}$	GGYKAADMW	0			34			0			0			11			0			**61**	n.d.	n.d.	0		
Rv1886c$_{215-223}$	KAADMWGPS	33			4			1			**103**	7E-09	0.8	17			18			21			34		
Rv1886c$_{219-227}$	MWGPSSDPA	6			24			0			**74**	n.d.	n.d.	7			3			0			0		
Rv1886c$_{222-230}$	PSSDPAWER	1			0			0			0			**53**	n.d.	n.d.	10			2			18		
Rv1886c$_{233-241}$	PTQQIPKLV	3			11			10			**86**	n.d.	n.d.	0			5			9			22		
Rv1886c$_{235-243}$	QQIPKLVAN	36			0			10			**60**	n.d.	n.d.	2			2			16			15		
Rv1886c$_{237-245}$	IPKLVANNT	0			0			5			0			0			**41**	n.d.	n.d.	16			0		
Rv1886c$_{239-247}$	**KLVANNTRL**	**81**	n.d.	n.d.	**73**	n.d.	n.d.	**60**	2E-08	0.7	**167**	1E-07	2.2	18			**43**	n.d.	n.d.	**40**			13		
Rv1886c$_{240-248}$	**LVANNTRLW**	21			**88**	n.d.	n.d.	2			**68**	5E-07	0.5	38			**50**	n.d.	n.d.	**50**	n.d.	n.d.	26		
Rv1886c$_{241-249}$	**VANNTRLWV**	**77**	5E-07	2.5	**58**	4E-06	0.3	**141**	3E-08	0.5	**171**	1E-07	0.8	3			**61**	6E-07	0.7	**49**	1E-08	1.9	**69**	3E-07	0.6
Rv1886c$_{242-250}$	*ANNTRLWVY*	5			32			19			**195**	2E-08	1.9	0			9			19			**113**	5E-09	0.7
Rv1886c$_{243-251}$	NNTRLWVYC	0			3			19			**89**	n.d.	n.d.	4			4			11			20		
Rv1886c$_{244-252}$	NTRLWVYCG	0			0			27			**49**	n.d.	n.d.	0			0			0			0		
Rv1886c$_{245-253}$	TRLWVYCGN	0			0			**45**	n.d.	n.d.	33			5			0			0			15		
Rv1886c$_{246-254}$	*RLWVYCGNG*	**45**	n.d.	n.d.	0			0			**65**	n.d.	n.d.	12			2			8			9		
Rv1886c$_{248-256}$	WVYCGNGTP	11			0			0			0			**55**	n.d.	n.d.	2			4			16		
Rv1886c$_{249-257}$	*VYCGNGTPN*	0			11			8			**80**	n.d.	n.d.	4			3			15			**59**	n.d.	n.d.
Rv1886c$_{251-259}$	CGNGTPNEL	20			2			0			**41**	n.d.	n.d.	0			0			0			28		
Rv1886c$_{254-262}$	GTPNELGGA	49	n.d.	n.d.	0			0			0			0			0			0			0		
Rv1886c$_{258-266}$	ELGGANIPA	47	n.d.	n.d.	0			0			0			0			0			0			0		
Rv1886c$_{260-268}$	*GGANIPAEF*	4			0			0			**70**	n.d.	n.d.	0			0			**57**	n.d.	n.d.	5		
Rv1886c$_{261-269}$	GANIPAEFL	**41**	n.d.	n.d.	0			0			0			0			0			16			0		
Rv1886c$_{264-272}$	*IPAEFLENF*	17			**48**	n.d.	n.d.	0			0			0			**47**	n.d.	n.d.	0			2		
Rv1886c$_{266-274}$	AEFLENFVR	12			1			0			0			**63**	n.d.	n.d.	0			10			13		
Rv1886c$_{268-276}$	FLENFVRSS	**77**	n.d.	n.d.	5			0			0			7			0			0			5		
Rv1886c$_{271-279}$	*NFVRSSNLK*	3			0			**101**	9E-07	0.4	0			**84**	3E-06	0.2	0			10			0		
Rv1886c$_{272-280}$	**FVRSSNLKF**	15			**81**	n.d.	n.d.	0			**94**	8E-06	1.9	**45**	8E-08	0.1	**114**	n.d.	n.d.	12			**76**	2E-08	1.4
Rv1886c$_{273-281}$	VRSSNLKFQ	5			23			0			0			14			10			7			**61**	n.d.	n.d.
Rv1886c$_{274-282}$	*RSSNLKFQD*	11			26			0			**104**	1E-05	1.6	11			3			14			**64**	n.d.	n.d.
Rv1886c$_{275-283}$	SSNLKFQDA	32			12			0			**64**	n.d.	n.d.	14			0			9			10		
Rv1886c$_{276-284}$	*SNLKFQDAY*	11			23			0			**124**	2E-07	1.6	18			0			14			**52**	n.d.	n.d.
Rv1886c$_{277-285}$	NLKFQDAYN	8			0			0			**123**	1E-05	0.4	9			0			0			7		
Rv1886c$_{278-286}$	*LKFQDAYNA*	17			0			0			**91**	n.d.	n.d.	0			0			9			**53**	n.d.	n.d.
Rv1886c$_{279-287}$	KFQDAYNAA	19			0			0			**113**	8E-06	0.7	0			0			0			0		
Rv1886c$_{282-290}$	DAYNAAGGH	4			14			8			18			**64**	n.d.	n.d.	12			29			16		
Rv1886c$_{284-292}$	*YNAAGGHNA*	16			0			10			**53**	n.d.	n.d.	32			0			**46**	n.d.	n.d.	12		
Rv1886c$_{285-293}$	NAAGGHNAV	39			0			27			2			28			16			**43**	n.d.	n.d.	5		
Rv1886c$_{286-294}$	*AAGGHNAVF*	31			34			15			**67**	n.d.	n.d.	0			9			**101**	2E-07	0.7	39		
Rv1886c$_{288-296}$	*GGHNAVFNF*	**40**	n.d.	n.d.	34			0			0			0			19			**52**	n.d.	n.d.	22		
Rv1886c$_{291-299}$	NAVFNFPPN	24			14			0			**70**	n.d.	n.d.	27			5			28			2		
Rv1886c$_{292-300}$	**AVFNFPPNG**	**69**	n.d.	n.d.	23			21			**101**	3E-06	0.3	**81**	8E-09	0.2	0			33			1		
Rv1886c$_{293-301}$	VFNFPPNGT	34			25			3			**113**	3E-05	1.2	3			0			33			0		
Rv1886c$_{295-303}$	NFPPNGTHS	14			0			11			**43**	n.d.	n.d.	0			0			26			0		
Rv1886c$_{298-306}$	*PNGTHSWEY*	0			0			0			**77**	n.d.	n.d.	9			8			0			**48**	n.d.	n.d.
Rv1886c$_{299-307}$	**NGTHSWEYW**	0			**42**	n.d.	n.d.	0			**83**	1E-06	1.6	35			4			**74**	4E-08	1.9	3		
Rv1886c$_{300-308}$	**GTHSWEYWG**	**43**	2E-06	0.3	2			0			**79**	3E-05	2.2	**71**	1E-05	0.2	3			**58**	3E-07	2.9	25		
Rv1886c$_{301-309}$	*THSWEYWGA*	21			0			0			**67**	n.d.	n.d.	**57**	n.d.	n.d.	2			0			6		
Rv1886c$_{303-311}$	SWEYWGAQL	31			**79**	n.d.	n.d.	0			0			0			1			0			0		
Rv1886c$_{306-314}$	*YWGAQLNAM*	11			**113**	8E-09	0.5	0			**63**	n.d.	n.d.	16			12			0			0		
Rv1886c$_{307-315}$	*WGAQLNAMK*	7			0			0			**67**	n.d.	n.d.	**153**	3E-07	2.4	0			0			5		
Rv1886c$_{309-317}$	AQLNAMKGD	10			29			0			**56**	n.d.	n.d.	0			0			0			3		

		A*02:01			A*24:02			A*30:01			A*30:02			A*68:01			B*07:02			B*58:01			C*07:01		
**Peptide ID**	**Sequence***	**Bind****	**Aff**	**O-rate**	**Bind**	**Aff**	**O-rate**	**Bind**	**Aff**	**O-rate**	**Bind**	**Aff**	**O-rate**	**Bind**	**Aff**	**O-rate**	**Bind**	**Aff**	**O-rate**	**Bind**	**Aff**	**O-rate**	**Bind**	**Aff**	**O-rate**
Rv1886c$_{310\text{-}318}$	**QLNAMKGDL**	**71**	n.d.	n.d.	**60**	n.d.	n.d.	10			0			0			**49**	n.d.	n.d.	0			17		
Rv1886c$_{312\text{-}320}$	NAMKGDLQS	14			**45**	n.d.	n.d.	6			0			15			6			0			20		
Rv1886c$_{313\text{-}321}$	**AMKGDLQSS**	**89**	2E-07	1.7	**42**	4E-04	0.3	**129**	2E-06	0.5	**47**	1E-07	2	0			9			0			23		
Rv1886c$_{314\text{-}322}$	*MKGDLQSSL*	16			22			14			**90**	2E-05	2.8	19			17			0			**63**	n.d.	n.d.
Rv1886c$_{315\text{-}323}$	KGDLQSSLG	10			4			20			13			**40**	n.d.	n.d.	6			0			25		

*Promiscuous epitopes are marked in bold or italic (italic - binding to >1 allele, bold - binding to >3 alleles. **Positive binding epitopes are marked in bold. Binding is reported as percent relative the binding of a positive control peptide, affinity is reported as an ED50 value (M), and off-rate is reported as a $t_{1/2}$ value (h), as described in Material and methods. Tetramers were constructed for the epitopes: A02-Rv1886c$_{\text{YLLDGLRAQ}}$, A02-Rv1886c$_{\text{FIYAGSLSA}}$, A02-Rv1886c$_{\text{KLVANNTRL}}$, A24-Rv1886c$_{\text{WYYQSGLSI}}$, A24-Rv1886c$_{\text{FLTSELPQW}}$, A24-Rv1886c$_{\text{IYAGSLSAL}}$, A3001-Rv1886c$_{\text{VANNTRLWV}}$, A3002-Rv1886c$_{\text{VANNTRLWV}}$, A68-Rv1886c$_{\text{LPQWLSANR}}$, A68-Rv1886c$_{\text{WGAQLNAMK}}$, B07-Rv1886c$_{\text{MPVGGQSSF}}$, B07-Rv1886c$_{\text{IYAGSLSAL}}$, B07-Rv1886c$_{\text{GPSLIGLAM}}$, B07-Rv1886c$_{\text{IPKLVANNT}}$ B58-Rv1886c$_{\text{QTYKWETFL}}$ and C07-Rv1886c $_{\text{ANNTRLWVY}}$.
