# Supplementary material for: A Broad Profile of Co-Dominant Epitopes Shapes the Peripheral Mycobacterium tuberculosis Specific CD8+ T-Cell Immune Response in South African Patients with Active Tuberculosis
Source: PLoS One. 2013 Mar 26;8(3):e58309. doi: 10.1371/journal.pone.0058309 (PMC3608651; doi:10.1371/journal.pone.0058309)
Supplement: Table S3 — Frequency of epitope-specific T cells identified by multimer staining. (PDF) [file pone.0058309.s009.pdf]

**Table S3.** Frequency of epitope-specific T cells identified by multimer staining*.

| Tetramer/Patient | 1265 | 1284 | 1288 | 1292 | 1421 | 1903 | 3104 | 3112 | 3245 | 3246 | 3282 | 3283 | 4063 | 5363 | 5364 | 5436 | 5437 | 5562 | 5905 | 6088 | 6089 | 7235 | 7237 | 7238 | 7606 | 7767 | 7769 | AVERAGE |
|---|---|---|---|---|---|---|---|---|---|---|---|---|---|---|---|---|---|---|---|---|---|---|---|---|---|---|---|---|
| A2-RV0288$_{IMYNYPAML}$ | | | | | | | | | | | | | | | | **0.8** | **1.0** | 0.1 | | | 0.0 | | | | | | | *0.5* |
| A2-RV0288$_{AMLGHAGDM}$ | | | | | | | | 0.2 | | | | | | | | **0.1** | | | | | 0.0 | | | | | | | *0.1* |
| A2-RV0288$_{MLGHAGDMA}$ | | | | | | | | 0.3 | | | | | | | | 0.1 | | | | | 0.0 | | | | | | | *0.1* |
| A2-Rv1886c$_{YLLDGLRAQ}$ | | | | | | | | | | | | | | | | 0.0 | **0.3** | 0.1 | | | 0.0 | | | | | | | *0.1* |
| A2-Rv1886c$_{KLVANNTRL}$ | | | | | | | | | | | | | 0.4 | | | **0.2** | **1.4** | 0.1 | | | 0.1 | | | | | 0.5 | | *0.4* |
| A2-Rv1886c$_{FIYAGSLSA}$ | | | | | | | | | | | | | 0.7 | | | **0.0** | 0.0 | 0.0 | | | **0.5** | | | | | 0.1 | | *0.2* |
| A2-RV3875$_{AMASTEGNV}$ | | | | | | | | | | | | | 0.2 | | | 0.1 | **0.4** | 0.2 | | | **0.2** | | | | | 0.0 | | *0.2* |
| A2-RV3875$_{LLDEGKQSL}$ | | | | | | | | | | | | | 0.6 | | | 0.0 | 0.0 | 0.2 | | | 0.0 | | | | | 0.0 | | *0.1* |
| A2-Rv2958$_{ALADLPVTV}$ | | | | | | | | | | | | | 0.2 | | | 0.0 | 0.0 | | | | 0.0 | | | | | | | *0.1* |
| A2-Rv2957$_{SIIIPTLNV}$ | | | | | | | | | | | | | 0.0 | | | 0.2 | 0.0 | | | | 0.0 | | | | | | | *0.1* |
| A2-Rv0447$_{VLAGSVDEL}$ | | | | | | | | | | | | | 0.2 | | | 0.0 | 0.0 | 0.0 | | | 0.0 | | | | | | | *0.0* |
| A24-RV0288$_{IMYNYPAML}$ | | | | | | | | | **0.4** | **0.3** | | | | | | | | | **0.4** | **0.2** | | | | | | | | *0.3* |
| A24-RV0288$_{MYNYPAMLG}$ | | | | | | | | | | | | | | | | | | | **0.4** | **0.5** | | | | | | | | *0.4* |
| A24-Rv1886c$_{WYYQSGLSI}$ | | | | | | | | | **0.2** | **0.5** | | | | | | | | | **1.1** | **1.1** | | | | | | | | *0.7* |
| A24-Rv1886c$_{FLTSELPQW}$ | | | | | | | | | **0.9** | **0.1** | | | | | | | | | **0.7** | **0.5** | | | | | | | | *0.5* |
| A24-Rv1886c$_{IYAGSLSAL}$ | | | | 0.2 | | | | | 0.0 | 0.0 | | | | | | | | | 0.1 | 0.0 | | | | | | | | *0.1* |
| A24-RV3875$_{AYQGVQQKW}$ | | | | 0.0 | | | | | 0.0 | **0.1** | | | | | | | | | 0.1 | 0.0 | | | | | | | | *0.0* |
| A24-RV3875$_{ELNNALQNL}$ | | | | 3.9 | | | | | **1.9** | **2.2** | | | | | | | | | **0.7** | **0.8** | | | | | | | | *1.9* |
| A24-Rv2958$_{KYIAADRKI}$ | | | | 0.2 | | | | | 0.0 | 0.0 | | | | | | | | | **0.3** | **0.4** | | | | | | | | *0.2* |
| A24-Rv2957$_{PYNLRYRVL}$ | | | | 1.2 | | | | | **0.3** | **0.1** | | | | | | | | | **0.9** | **1.2** | | | | | | | | *0.7* |
| A24-Rv0447$_{KYIFPGGLL}$ | | | | 0.3 | | | | | 0.0 | 0.0 | | | | | | | | | | **0.1** | | | | | | | | *0.1* |
| A3001-RV0288$_{QIMYNYPAM}$ | | **0.4** | | | | **0.3** | | **0.3** | | | | 0.2 | | **0.2** | | | | | | **0.2** | | | | | | | **0.8** | *0.3* |
| A3001-RV0288$_{LVRAYHAMS}$ | | **0.1** | | | | | | **0.2** | | | | **0.5** | | **0.2** | | | | | | **0.1** | | | | | | | **0.4** | *0.3* |
| A3001-Rv1886c$_{VANNTRLWV}$ | 0.0 | 0.0 | | | | 0.0 | | | | | | 0.0 | | 0.0 | 0.0 | | | | | 0.0 | | | | | | | 0.0 | *0.0* |
| A3001-Rv2957$_{IVLVRRWPK}$ | | **0.3** | | | | **0.5** | | 0.1 | | | | **0.3** | | 0.1 | | | | | | **0.1** | | | | | | | **0.5** | *0.3* |
| A3002-RV0288$_{QIMYNYPAM}$ | | **0.2** | | | | **0.2** | | **0.3** | | | | **0.2** | | 0.2 | | | | | | **0.2** | | | | | | | **0.9** | *0.3* |
| A3002-RV0288$_{IMYNYPAML}$ | | **0.2** | | | | **0.1** | | **0.7** | | | | **0.2** | | 0.1 | | | | | | 0.1 | | | | | | | **1.4** | *0.4* |
| A3002-RV0288$_{AMEDLVRAY}$ | | **0.2** | | | | **0.3** | | 0.2 | | | | **0.3** | | **0.1** | | | | | | **0.1** | | | | | | | **0.4** | *0.2* |
| A3002-Rv1886c$_{VANNTRLWV}$ | 0.0 | 0.0 | | | | 0.0 | | | | | | 0.0 | | 0.0 | 0.0 | | | | | 0.0 | | | | | | | 0.0 | *0.0* |
| A3002-RV3875$_{AMASTEGNV}$ | **0.1** | 0.1 | | | | **0.3** | | | | | | 0.1 | | 0.0 | 0.0 | | | | | 0.0 | | | | | | | 0.1 | *0.1* |
| A3002-Rv2958$_{SARLAGIPY}$ | | **0.3** | | | | **0.3** | | **0.5** | | | | **0.8** | | **0.2** | | | | | | **0.2** | | | | | | | **0.4** | *0.4* |
| A3002-Rv0447$_{RMWELYLAY}$ | | **0.1** | | | | **0.1** | | 0.1 | | | | **0.3** | | **0.1** | | | | | | **0.1** | | | | | | | 0.1 | *0.1* |
| A68-RV0288$_{HAMSSTHEA}$ | | 0.1 | 0.5 | | | | | | | | **1.4** | | | | | **0.3** | | | | | | | | | | | | *0.6* |
| A68-RV0288$_{ANTMAMMAR}$ | | 0.1 | 0.3 | | | | | | | | **0.4** | | | | | **0.1** | | | | | | | | | | | | *0.2* |
| A68-Rv1886c$_{LPQWLSANR}$ | | 0.0 | 0.1 | | | | | | | | 0.0 | | | | | 0.2 | | | | | | | | | | | | *0.1* |
| A68-Rv1886c$_{WGAQLNAMK}$ | | **0.2** | 0.1 | | | | | | | | **0.8** | | | | | **0.2** | | | | | | | | | | | | *0.3* |
| A68-Rv2958$_{AAPEPVARR}$ | | 0.0 | 0.0 | | | | | | | | **0.3** | | | | | 0.2 | | | | | | | | | | | | *0.1* |
| A68-Rv2957$_{LVYGDVIMR}$ | | 0.0 | 0.0 | | | | | | | | 0.0 | | | | | 0.0 | | | | | | | | | | | | *0.0* |
| A68-Rv0447$_{AASAAIANR}$ | | 0.0 | 0.0 | | | | | | | | 0.1 | | | | | 0.0 | | | | | | | | | | | | *0.0* |
| B07-Rv1886c$_{IPKLVANNT}$ | | | | | | **0.4** | | | 0.0 | | | 0.0 | | | | | | | | | | | | | | | | *0.1* |
| B07-Rv1886c$_{IYAGSLSAL}$ | | | | | | **0.2** | | | 0.0 | | | 0.0 | | | | | | | | | | | | | | | | *0.1* |
| B07-Rv1886c$_{GPSLIGLAM}$ | | | | | | 0.1 | | | | | | 0.0 | | | | | | | | | | | | | | | | *0.1* |
| B07-Rv1886c$_{MPVGGGSSF}$ | | | | | | 0.1 | | | | | | 0.0 | | | | | | | | | | | | | | | | *0.0* |
| B58-Rv1886c$_{QTYKWETFL}$ | | | 0.0 | | 0.0 | | 0.0 | | | 0.0 | | | | | 0.0 | **1.1** | | | 0.1 | **0.1** | | | | | 0.0 | | 0.1 | *0.1* |
| C07-Rv1886c$_{ANNTRLWVY}$ | | **0.4** | | 0.0 | | | | | 0.0 | **0.1** | | | | **0.5** | | | | | | | **0.3** | **0.1** | **0.1** | **0.1** | | | **0.9** | *0.2* |

*PBMCs from individuals with TB were incubated with MHC-matched MHC class I-TB multimers and stained for T-cell markers. Results are reported as percent multimer-positive events in the CD3+CD8+ T-cell population; negative gating was performed to exclude CD4+ T cells. Negative multimers were included to decipher the background staining. For bold points it was possible to retrieve the phenotype as well as data regarding the degranulation marker CCR7 and the survival marker CD127 of the antigen-specific T-cells.
